# The N-terminus of Spt16 anchors FACT to MCM2–7 for parental histone recycling

**DOI:** 10.1093/nar/gkad846

**Published:** 2023-10-18

**Authors:** Xuezheng Wang, Yuantao Tang, Jiawei Xu, He Leng, Guojun Shi, Zaifeng Hu, Jiale Wu, Yuwen Xiu, Jianxun Feng, Qing Li

**Affiliations:** State Key Laboratory of Protein and Plant Gene Research, School of Life Sciences and Peking-Tsinghua Center for Life Sciences, Peking University, Beijing, 100871, China; Academy for Advanced Interdisciplinary Studies, Peking University, Beijing, 100871, China; State Key Laboratory of Protein and Plant Gene Research, School of Life Sciences and Peking-Tsinghua Center for Life Sciences, Peking University, Beijing, 100871, China; State Key Laboratory of Protein and Plant Gene Research, School of Life Sciences and Peking-Tsinghua Center for Life Sciences, Peking University, Beijing, 100871, China; State Key Laboratory of Protein and Plant Gene Research, School of Life Sciences and Peking-Tsinghua Center for Life Sciences, Peking University, Beijing, 100871, China; Academy for Advanced Interdisciplinary Studies, Peking University, Beijing, 100871, China; State Key Laboratory of Protein and Plant Gene Research, School of Life Sciences and Peking-Tsinghua Center for Life Sciences, Peking University, Beijing, 100871, China; State Key Laboratory of Protein and Plant Gene Research, School of Life Sciences and Peking-Tsinghua Center for Life Sciences, Peking University, Beijing, 100871, China; State Key Laboratory of Protein and Plant Gene Research, School of Life Sciences and Peking-Tsinghua Center for Life Sciences, Peking University, Beijing, 100871, China; Academy for Advanced Interdisciplinary Studies, Peking University, Beijing, 100871, China; State Key Laboratory of Protein and Plant Gene Research, School of Life Sciences and Peking-Tsinghua Center for Life Sciences, Peking University, Beijing, 100871, China; State Key Laboratory of Protein and Plant Gene Research, School of Life Sciences and Peking-Tsinghua Center for Life Sciences, Peking University, Beijing, 100871, China; Academy for Advanced Interdisciplinary Studies, Peking University, Beijing, 100871, China

## Abstract

Parental histone recycling is vital for maintaining chromatin-based epigenetic information during replication, yet its underlying mechanisms remain unclear. Here, we uncover an unexpected role of histone chaperone FACT and its N-terminus of the Spt16 subunit during parental histone recycling and transfer in budding yeast. Depletion of Spt16 and mutations at its middle domain that impair histone binding compromise parental histone recycling on both the leading and lagging strands of DNA replication forks. Intriguingly, deletion of the Spt16-N domain impairs parental histone recycling, with a more pronounced defect observed on the lagging strand. Mechanistically, the Spt16-N domain interacts with the replicative helicase MCM2–7 and facilitates the formation of a ternary complex involving FACT, histone H3/H4 and Mcm2 histone binding domain, critical for the recycling and transfer of parental histones to lagging strands. Lack of the Spt16-N domain weakens the FACT-MCM interaction and reduces parental histone recycling. We propose that the Spt16-N domain acts as a protein-protein interaction module, enabling FACT to function as a shuttle chaperone in collaboration with Mcm2 and potentially other replisome components for efficient local parental histone recycling and inheritance.

## Graphical Abstract

**Graphical Abstract:**
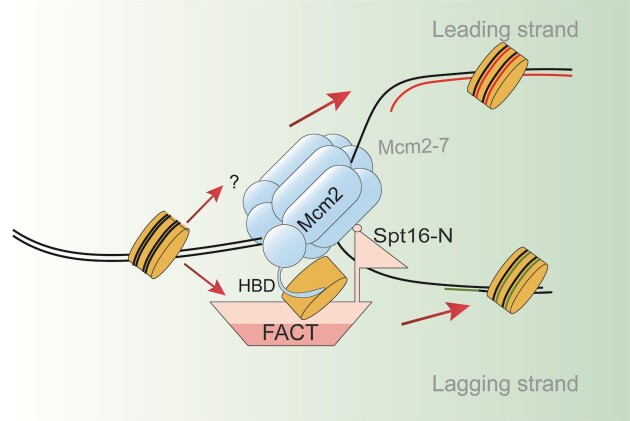

## Introduction

In eukaryotic cells, DNA is packaged into chromatin (1). The basic repeating unit of chromatin is the nucleosome, which is composed of approximately 147 base pairs (bp) of DNA wrapped around a histone octamer (2). The histone octamer consists of a tetramer of histones (H3/H4)_2_ and two dimers of H2A-H2B histones. Histones are modified post-translationally, and these post-translational modifications (PTM) govern different cellular events including DNA replication, DNA repair, gene transcription and high-order chromatin organization (3–6). Certain PTMs can be inherited during cell division (7–10). Growing evidence suggests that parental histones are recycled onto newly synthesized DNA strands in the same position they occupied previously (11–13). Thus, the transfer of parental histones during chromatin replication plays a pivotal role in preserving epigenetic memory (10,14).

During DNA replication, the replicative helicase MCM2–7 unwinds double-stranded DNA, generating template strands for DNA synthesis (15,16). In the context of chromatin, the nucleosome located ahead of the replication fork needs to be disassembled (17,18), allowing for the transfer of parental histones to the daughter strands. Studies have demonstrated that histones bound to chromatin with specific modifications, such as tri-methylation of H3 lysine 4 (H3K4me3), are recycled onto newly synthesized chromatin (19). Recent advancements in methodology have enabled the identification of recycled parental histones on specific daughter strands (leading or lagging strands) in both budding yeast and mammalian cells (19–21). Additionally, several components of the replisome actively participate in the transfer of parental histones. For example, in budding yeast, Dpb3 and Dpb4, two subunits of DNA polymerase ϵ (Pol ϵ), facilitate the transfer of parental histones to the leading strand, while the Mcm2-Ctf4-DNA polymerase α (Pol α) axis aids in the transfer of parental histones to the lagging strand (17,19,22). Although some of these replisome components possess a histone binding motif that can act as a co-chaperone, the precise mechanisms by which these replisome components function during parental histone recycling remain to be fully elucidated.

The histone chaperone FACT is essential for chromatin replication (18). It has the ability to bind to both nucleosomal and free histones, exhibiting disassembly and reassembly capabilities (23–27). Additionally, FACT can induce global accessibility of nucleosomes without ATP hydrolysis (26). Furthermore, FACT is found in the replisome complex and interacts with multiple replisome components, including the MCM2–7 helicase (28,29), DNA pol α (30), RPA (31,32) and the fork protection complex (33,34). Since the histone binding domain of Mcm2 is required for parental histone recycling, it has been proposed that the cooperation between FACT and Mcm2 is important for this purpose (28). However, evidence supporting the involvement of FACT in parental histone recycling *in vivo* remains lacking; and further, the manner by which FACT cooperates with Mcm2 in parental histone transfer remains to be determined.

The FACT complex in budding yeast consists of Spt16 and Pob3 (30). Structural and functional studies have revealed that FACT contains multiple histone binding domains. For instance, the middle domain of both Spt16 and Pob3 contains a tandem repeat of pleckstrin homology (PH) domains that bind to histone H3/H4 (35,36). The C-terminal domain of Spt16 can bind to histone H2A–H2B (27,37), and the N-terminal domain of Spt16 is a peptidase homology domain without enzyme activity (38–40). While the N-terminal domain can bind directly to histone H3/H4 *in vitro*, it has been shown that N-terminal deletion of Spt16 does not reduce the affinity of FACT for the nucleosome (38,39), suggesting that the Spt16-N domain may play other roles in chromatin transactions. Recently, we found that the Spt16-N domain functions as a protein–protein interaction module that contacts with multiple factors involved in DNA replication and gene transcription (34). Moreover, the interactions of FACT with several replisome components, including the fork protection complex subunits (Mrc1–Tof1–Csm3) and DNA Pol α, are reduced or abolished in cells lacking the Spt16-N domain (34). This observation has been confirmed by independent analysis demonstrating that the Spt16-N domain interacts with the C-terminus of Tof1 and that this interaction is required for the positioning of FACT with the fork protection complex at replication forks (33). Thus, the interaction between FACT and replisome factors mediated by the Spt16-N domain may provide a mechanism to coordinate FACT with Mcm2 during nucleosome assembly.

Here, we demonstrate the involvement of FACT in the recycling of parental histones to both the leading and lagging strands during DNA replication forks. Surprisingly, when the Spt16-N domain is deleted, there is an apparent disruption in the transfer of parental histones, with a more pronounced effect on the lagging strand compared to the corresponding leading strand in yeast cells. Mechanistically, the Spt16-N domain is essential for the interaction between FACT and the MCM2–7 complex, which regulates the formation of FACT–H3/H4 FACT–Mcm2 ternary complex. Collectively, our findings provide *in vivo* evidence supporting the role of FACT in parental histone recycling on both the leading and lagging strands, highlighting an unexpected function for the Spt16-N domain in this process.

## Materials and methods

### Yeast strains, media, antibodies and reagents

The budding yeast strains used in this study were in the isogenic W303-1 background and are listed in Supplementary Table S1. Standard protocols were used for yeast culture and the generation of mutant yeast strains. Antibodies and reagents used in this study are listed in Supplementary Table S2 and S3, respectively.

### Yeast cell synchronization and culture

*MATa* yeast cells were cultured in YPD medium at 30°C to a density of OD_600_ = 0.4–0.5. A 1:1000 volume of 5 mg/ml alpha factor (Chinese Peptide MATE-001A) was added to the medium. After a 2-h incubation at 25°C, cells were released into fresh YPD medium containing 0.4 mg/ml bromodeoxyuridine (BrdU) for eSPAN (enrichment and sequencing of protein associated nascent DNA) analysis.

### Chromatin immunoprecipitation (ChIP)

ChIP was performed as described previously (32). Briefly, yeast cells were synchronized at G1 phase, then released into YPD medium for 30 and 40 min at 25°C to allow for the accumulation of early S phase cells. The collected yeast cells were crosslinked with 1% freshly made formaldehyde at 25°C for 20 min, and subsequently quenched with 125 mM glycine for 5 min. For HA and PCNA ChIP, the crosslinked cells were first lysed using glass beads in ChIP lysis buffer (50 mM HEPES pH 8.0, 150 mM NaCl, 2 mM EDTA, 1% Triton X-100 and 0.1% sodium deoxycholate). Lysates were then fragmented by sonication (25 cycles: 20 s on and 30 s off, Bioruptor® Next gen Diagenode) and clarified. An aliquot of each cell lysate was saved as input, and the remaining lysates were subjected to immunoprecipitation using antibodies against HA (Roche 12ca5 11583816001) or PCNA (a kind gift from Zhiguo Zhang's lab) with rotation at 4°C. The next day, immunoprecipitated samples were incubated with Protein G beads for 2 h, which were washed and then boiled to release ChIP DNA. For Spt16-TAP ChIP, IgG beads were incubated with the lysate for 3 h, subsequently washed extensively and ChIP DNA was eluted. ChIP DNA was analyzed by quantitative PCR and normalized to input. The mean and standard deviation of three biological replicates are shown. PCR primers are listed in Supplementary Table S4.

### eSPAN (enrichment and sequencing of protein-associated nascent DNA)

The eSPAN assay was adapted as described previously with minor modifications (19). Briefly, yeast cells were synchronized at G1 phase with 5 μg/ml α-factor (Chinese Peptide Company) at 25°C and subsequently released into fresh YPD media containing 0.4 mg/ml 5-bromo-2-deoxyuridine (BrdU) (Sigma-Aldrich, B5002-5G), an analog of thymidine that is incorporated into nascent DNA during DNA synthesis in S phase, for 40 min at 23°C. After crosslinking with 1% (w/v) paraformaldehyde (Sigma-Aldrich, P6148-1KG) at 25°C for 20 min, followed by quenching with 125 mM glycine (Amresco, 0167-5KG) at 25°C for 5 min.

For histone ChIP, the resulting cells were then pelleted and washed twice with cold TBS buffer (0.1 mM PMSF freshly added) and once with cold Buffer Z (1.2 M sorbitol, 50 mM Tris–HCl pH 7.4). The cells were resuspended in Buffer Z (10 mM β-mercaptoethanol freshly added) and digested by adding approximately 123 μg/ml zymolase (nacalai tesque, 07665–84), followed by incubation at 28°C and 100 rpm for approximately 35 min. The efficiency of digestion was assessed by measuring the OD_600_ in 1% SDS, which should decrease to less than 10% of the pre-digestion value. The spheroplasts were then collected and the supernatant was carefully aspirated. The pellet was gently resuspended in NP buffer (1 M sorbitol, 50 mM NaCl, 10 mM Tris–HCl pH 7.4, 5 mM MgCl_2_, 1 mM CaCl_2_, with 0.5 mM Spermidine, 0.007% (v/v) β-mercaptoethanol and 0.075% (v/v) NP-40 (Thermo, 28324) freshly added). The resuspended pellet was divided into four equal parts, with each part containing 400 μl. To digest the chromatin mainly into mono- and di-nucleosomes, the appropriate amount of MNase (Worthington, LS004797) (∼ 1.5 μl 7.5 U/μl MNase stock for each reaction) was added to each part, and the reaction mixtures were incubated at 37°C for 20 min. The reaction was terminated by the addition of 10 mM EDTA, and a one-fourth volume of 5 × ChIP buffer (250 mM HEPES–KOH pH 7.5, 700 mM NaCl, 5 mM EDTA pH 8.0, 5% (v/v) Triton X-100, 0.5% (w/v) sodium deoxycholate, with 5 mM PMSF, 1.25 mg/ml pefabloc, 5 mg/ml bacitracin and 5 mM benzamidine freshly added) was added to the reaction mixtures, followed by 30 min of incubation on ice. The lysate was spun down twice at 10 800 rpm for 5 min and 15 min, respectively, at 4°C. The supernatant was collected and used for DNA extraction.

A small fraction of the digested chromatin was subjected to immunoprecipitation using antibodies against BrdU followed by strand-specific sequencing (BrdU-IP-ssSeq). The majority of digested chromatin was subjected to chromatin immunoprecipitation (ChIP) using antibodies against two different histone modifications, acetylation of lysine 56 of H3 (H3K56ac, a mark on new histones; kind gift from Zhiguo Zhang's lab) and tri-methylation of lysine 4 of H3 (H3K4me3, a parental histone mark, Abcam ab8580). The DNA was reverse crosslinked in elution buffer (10 mM Tris–HCl pH 8.0, 10 mM EDTA, 1% SDS, 150 mM NaCl, 5 mM freshly added DTT) and recovered with phenol–chloroform.

Subsequently, ChIP DNA was denatured into single-stranded DNA (ssDNA) and subjected to immunoprecipitation with BrdU antibodies to enrich newly synthesized DNA as described previously (32). The total nucleosomal or ChIP DNA was denatured for 10 min at 100°C and then chilled on ice for a further 10 min. A nine-fold volume of BrdU-IP buffer (PBS with 0.0625% Triton X-100 (v/v)) was added to the DNA sample containing 6.7 μg/ml tRNA and 0.17 μg/ml anti-BrdU antibody (BD 555627) and rotated for 2 h at 4°C. Protein G Sepharose® resin (GE Healthcare 17061805) was then added and incubated for 2 h at 4°C to allow for antibody binding. DNA was eluted with 1 × TE buffer containing 1% SDS for 15 min at 65°C and purified using a MinElute PCR purification kit (Qiagen 28006). ChIP and total nucleosomal DNA were subjected to eSPAN and BrdU-IP, respectively. DNA from all steps was prepared using the Accel-NGS 1S Plus kit (Swift 10096) or as described previously (32).

At least two independent biological repeats were performed for each experiment, and similar results were obtained. The raw sequence data reported in this paper have been deposited in the Genome Sequence Archive (41) in the National Genomics Data Center (42), China National Center for Bioinformation/Beijing Institute of Genomics, Chinese Academy of Sciences (GSA: CRA006313) and are publicly accessible at https://ngdc.cncb.ac.cn/gsa.

### Sequence mapping and data analysis

DNA samples were sequenced using the Illumina HiSeq X™ Ten system. Data were mapped to the *S. cerevisiae* reference genome (sacCer3) using the Bowtie2 software. Only consistent pair-end reads were used for further analysis. The reads mapped to Watson and Crick strands were separated using the in-house Perl program. Read coverage was calculated with a step size of 10 bp and normalized to total reads. BrdU-enriched regions were called by the MACS2 software. The histone modifications eSPAN sequence reads were separated and mapped to the Watson and Crick strands. To minimize the BrdU-incorporation difference, the reads were normalized to the corresponding reads from the MNase-BrdU-IP-Seq dataset.

To calculate the eSPAN bias between the leading and lagging strands, the log_2_ ratios of the Watson strand sequence reads over the Crick strand sequence reads in the same region surrounding the early replication origins in BrdU-IP were calculated using normalized eSPAN data. The replication origin information was taken from a previous report (32). Nucleosome positions were called using the statistics pipeline DANPOS (43). The average bias of each side was calculated using a 10-bp step size in the 15-kb DNA region surrounding early replication origins. The BrdU-incorporation level was calculated in the same region. This calculation, termed eSPAN bias, reflects the relative amount of a modified histone at the leading or lagging strand of DNA replication forks.

The relative amount of histone modifications in ±30 nucleosomes around the early replication origin in the eSPAN data was also calculated. The value of nucleosomes around the ACS on the leading or lagging strand was called by DANPOS. The log_2_ ratio of the nucleosome value of the Watson or Crick strand at the same position flanked by the ACS sites was calculated around each early DNA replication origin. Each row represents one origin, and each square represents the ratio of one nucleosome.

To determine the parental histone recycling rate, the ratio of total eSPAN sequence reads surrounding the ACS to the total MNase-BrdU-IP-Seq reads in the same region was calculated to measure the efficiency of parental histone recycling on nascent strands; this was termed eSPAN density. To avoid the noise caused by the low BrdU-incorporation level, only the well-replicated DNA region (2 kb around the early replication origins) was used for analysis. To compare the parental histone recycling rate between the leading and lagging strands, the eSPAN density was calculated using eSPAN sequence reads separated into Watson and Crick strands. Statistical significance was evaluated based on the Mann–Whitney *U* test.

### Tandem affinity purification

Yeast cells were harvested and resuspended in IP buffer (25 mM Tris pH 8.0, 100 mM NaCl, 1 mM EDTA, 10 mM MgCl_2_, 0.01% NP-40, 1 mM DTT) containing protease inhibitors (1 mM PMSF (Sigma P7626), 1 mM Benzamindine (Sigma B6506) and 1 mM Pefabloc (Roche 11429876001)) and 15 KU/ml DNase I (Sigma DN25). The resuspended cells were frozen in liquid nitrogen and ground in a Freezer/Mill grinder (SPEX). The lysates were clarified by centrifugation and incubated with IgG Sepharose® resin (GE Healthcare 17096902) for 2 h at 4°C, which were subsequently washed in IP buffer, and the proteins were eluted by incubation with TEV enzyme for 2 h at 16°C. Calmodulin affinity resin (Agilent 214303) in Calmodulin binding buffer (10 mM Tris–HCl pH 8.0, 100 mM NaCl, 1 mM magnesium acetate, 1 mM imidazole, 2 mM CaCl_2_, 0.01% NP-40, 1 mM DTT) was added and incubated for 2 h to allow for protein binding. The proteins were resolved by SDS-PAGE.

Spt16-TAP was purified for the *in vitro* pull-down assay as described above, with the exception that IP buffer containing 500 mM NaCl was used for extensive washing to remove the majority of FACT-associated proteins. The bound FACT complex was eluted from the IgG beads by incubation with TEV enzyme for 3 h at 16°C.

### Flag-tagged protein purification

Yeast cells were harvested and resuspended in Flag-IP buffer (25 mM Tris pH 8.0, 100 mM KAc, 1 mM EDTA, 10 mM MgCl_2_, 0.01% NP-40, 1 mM DTT) containing protease inhibitors (1 mM PMSF (Sigma P7626), 1 mM Benzamindine (Sigma B6506) and 1 mM Pefabloc (Roche 11429876001)). The resuspended cells were frozen in liquid nitrogen and ground in a Freezer/Mill grinder (SPEX). The lysates were clarified by centrifugation and incubated with ANTI-FLAG® M2 Affinity Gel (Sigma A2220) for 2 h at 4°C, which was then washed three times with Flag-IP buffer. The proteins were resolved by SDS-PAGE.

For pellet Flag-IP, the supernatants and pellets were clarified by centrifugation. The pellets were resuspended in Pellet Flag-IP buffer (25 mM Tris pH 8.0, 600 mM KAc, 1 mM EDTA, 10 mM MgCl_2_, 0.01% NP-40, 1 mM DTT) for 30 min. The clear lysates were clarified by centrifugation and incubated with ANTI-FLAG® M2 Affinity Gel (Sigma A2220) for 2 h at 4°C, which was then washed three times with Pellet Flag-IP buffer. The proteins were resolved by SDS-PAGE.

### GFP nanobody-based purification

To purify GFP-tagged proteins, cells were treated in the same manner as for tandem affinity purification but were bound to homemade GFP nanobody beads. The beads were washed extensively with IP buffer containing 500 mM NaCl and then incubated in IP buffer containing 100 mM NaCl. The protein-bound beads were used directly in the *in vitro* pull-down assay. At least two independent biological repeats were performed for each experiment, and similar results were obtained.

### Recombinant protein purification

Recombinant *Saccharomyces cerevisiae* histones H3 and H4 were expressed and purified as previously described (44). Briefly, yeast H3/H4 was engineered into a bicistronic pETDuet vector (Novagen). The histones were produced by co-expression in the BL21(DE3) CodonPlus RIL strain of *Escherichia coli*, induced by 0.4 mM IPTG for 3 h at 37°C. Harvested cells were ruptured by pressure in buffer containing 20 mM Tris–HCl pH 7.5, 500 mM NaCl, 10 mM β-mercaptoethanol. The clarified lysates were loaded onto HiTrap™ Heparin HP affinity columns (GE Healthcare 17040701), and a 0.5–2.0 M NaCl gradient was used to elute the bound proteins. The purified H3/H4 fractions were combined and analyzed by SDS-PAGE.

The GST-tagged Mcm2HBD (residues 1–200) was purified as previously described (32). Briefly, the Mcm2HBD coding sequence was inserted into the pGEX4T-1 vector, and protein expression in the BL21(DE3) CodonPlus RIL strain of *E. coli* was induced by 0.4 mM IPTG for 3 h at 37°C. Harvested cells were ruptured by pressure in lysis buffer (TBS containing 1% Triton X-100 and protease inhibitors (1 mM DTT, 1 mM PMSF, 1 mM DTT, 1 mM Benz)). The lysates were clarified by centrifugation and incubated with Glutathione Sepharose® resin (GE Healthcare 17513202), which was then washed with TBST buffer (TBS + 1% Triton X-100) containing 1 mM DTT and PMSF. The bound proteins were eluted using 100 mM l-glutathione buffer containing 1 mM DTT (Sigma G4251) and dialyzed in TBS buffer with 10% glycerol and 1 mM DTT.

### Immunoblotting

The samples were resuspended in SDS buffer, boiled at 100°C for 3 min and separated on homemade 10% or 15% SDS-PAGE gels at 120 V for 90 min. The proteins were then transferred to nitrocellulose membrane (GE Healthcare 10600002) at 0.25 A for 90 min. Subsequently, the membranes were probed with antibodies against histone H3 (Abcam ab1791 and EASYBIO BE3015), histone H4 (EASYBIO BE3194), H3K4me3 (Abcam ab8580), Flag tag (Sigma F1804), Mcm2 (a kind gift from Huiqiang Lou's lab), Pob3 (homemade), CBP (EASYBIO BE2068) and Spt16 (a kind gift from Tim Formosa's lab). Protein intensities were quantitated using the ImageJ (1.51i) software.

### *In vitro* pull-down assay

Purified proteins were quantitated using the Bradford assay (Bio-Rad 5000205). For GST pull-down, proteins (0.5 μg GST and 2 μg GST-Mcm2HBD) were bound to Glutathione Sepharose® resin and then washed three times in buffer A150 (25 mM Tris pH 7.5, 1 mM EDTA, 0.01% NP-40, 150 mM NaCl). For histone pull-down, 0.1, 0.2 and 0.4 μg histone H3/H4 in 0.4 ml buffer A150 was incubated with the resin for 6 h while rotating at 4°C and then washed three times in buffer A150. The bound proteins were resolved by SDS-PAGE and visualized by coomassie brilliant blue (CBB) staining.

For FACT pull-down, 0.2, 0.4, 0.8 and 1.6 μg wild-type FACT or Spt16-ΔN FACT in 0.8 ml buffer A150 was incubated with the resin for 6 h at 4°C and then washed three times in buffer A150. The bound proteins were resolved by SDS-PAGE and visualized by CBB staining.

For GST-Mcm2HBD pull-down shown in Supplementary Figure S7B and C, 2 μg GST-Mcm2HBD was bound to Glutathione Sepharose® resin, incubated with 0.4 μg histone H3/H4 in 0.4 ml buffer A150 for 6 h at 4°C, and then washed three times in buffer A150. Subsequently, 0.2, 0.4, 0.8 and 1.6 μg wild-type FACT or Spt16-ΔN FACT in 0.8 ml buffer A150 was incubated with this resin overnight at 4°C and then washed three times with buffer A150. The bound proteins were resolved by SDS-PAGE and visualized by CBB staining.

For *in vitro* histone capture, 0, 0.68, 0.90,1.35 and 2.0 μg wild-type FACT or Spt16-ΔN FACT in 0.8 ml buffer A150 was incubated with 400 ng histone H3/H4 overnight at 4°C to form the wild-type or Spt16-ΔN FACT–H3/H4 complex. Subsequently, 2 μg GST-Mcm2HBD was bound to Glutathione Sepharose® resin, incubated with the wild-type or Spt16-ΔN FACT–H3/H4 complex for 6 h, and then washed three times in buffer A150. The bound proteins were resolved by SDS-PAGE and visualized by CBB staining.

At least three independent biological repeats were performed for each experiment, and similar results were obtained. The quantitated results were from three independent experiments.

### Bimolecular fluorescence complementation (BiFC) assay

The BiFC assay was performed as previously described (45) using split YFP. The Vc155 was tagged into the *MCM2* gene locus in cells, and the Vn173 was fused to histone H3 driven by a *GAL1* promoter in the pRS426 plasmid. The pRS426 (*GAL1-HHT2-Vn173-HHF2*) were transformed into yeast cells with indicated backgrounds for BiFC analysis. Mcm5-Vn173-expressing yeast cells were used as a control. Cells were cultured to OD_600_ = 0.4 in SC media, the expression of H3-Vn173 were induced by 2% galactose for 2.5 h at 25°C. The resulted yeast cells were collected, washed with water and subsequently resuspended in PBS. Resuspended cells were spread on a petri dish and covered with 1% agarose gel. Images were captured using an Andor Dragonfly High Speed Confocal Microscope system (Andor Dragonfly 200) with a 514-nm laser to excite fluorescence, which was collected by a Fusion acquisition system and quantitated using the Fiji ImageJ 1.53C software. During the statistical analysis, we distinguished the yeast cell cycle stages based on the yeast morphology and counted the YFP signals in S-phase cells only. A paired Student's *t*-test was used to calculate the *P* value (n.s., not significant, *0.01 < *P* value ≤ 0.05, **0.001 < *P* value ≤ 0.01, ***P value ≤ 0.001).

## Results

### Spt16 participates in both the deposition of new H3/H4 and the transfer of parental H3/H4 to both replicating DNA strands

Using the separation-of-function mutant, *spt16-m (SPT16-K692R693AA)*, we previously demonstrated that FACT plays a role in the deposition of new H3/H4 onto replicating DNA (46). In addition, it has been proposed that FACT collaborates with Mcm2 to capture H3/H4 released from chromatin for the recycling of parental histones during DNA replication (28). Given that Mcm2 possesses a histone binding motif (HBD) and participates in the transfer of parental histone to the lagging strand, we aimed to investigate whether FACT is also involved in parental histone recycling and transfer at the replicated DNA strands in budding yeast cells. To achieve this, we employed eSPAN (enrichment and sequencing of protein-associated nascent DNA), a method that allows monitoring of the distribution of both parental and newly synthesized histones on the leading and lagging strands at the replicated regions (19,22,47). Considering FACT’s role in new histone deposition, we assessed the relative amount of two histone modifications, tri-methylation at lysine 4 of H3 (H3K4me3, a mark on parental histones) and acetylation at lysine 56 of H3 (H3K56ac, a mark on new histones) on the leading and lagging strands at the replicated regions under various conditions compromising FACT function.

Briefly, yeast cells were synchronized at G1 phase and subsequently released into fresh YPD media containing 5-bromo-2-deoxyuridine (BrdU), an analog of thymidine that is incorporated into nascent DNA during DNA synthesis in S phase. After crosslinking with paraformaldehyde, the chromatin was digested using micrococcal nuclease (MNase), which digests DNA between nucleosomes. A small fraction of digested chromatin was processed for immunoprecipitation using antibodies against BrdU followed by strand-specific sequencing (BrdU-IP-ssSeq). The majority of digested chromatin was subjected to chromatin immunoprecipitation (ChIP) using antibodies against H3K4me3 or H3K56ac. Subsequently, the ChIPed DNA was denatured into single-stranded DNA (ssDNA) and subjected to immunoprecipitation using BrdU antibodies to enrich newly synthesized DNA, followed by strand-specific deep sequencing.

**Figure 1.**
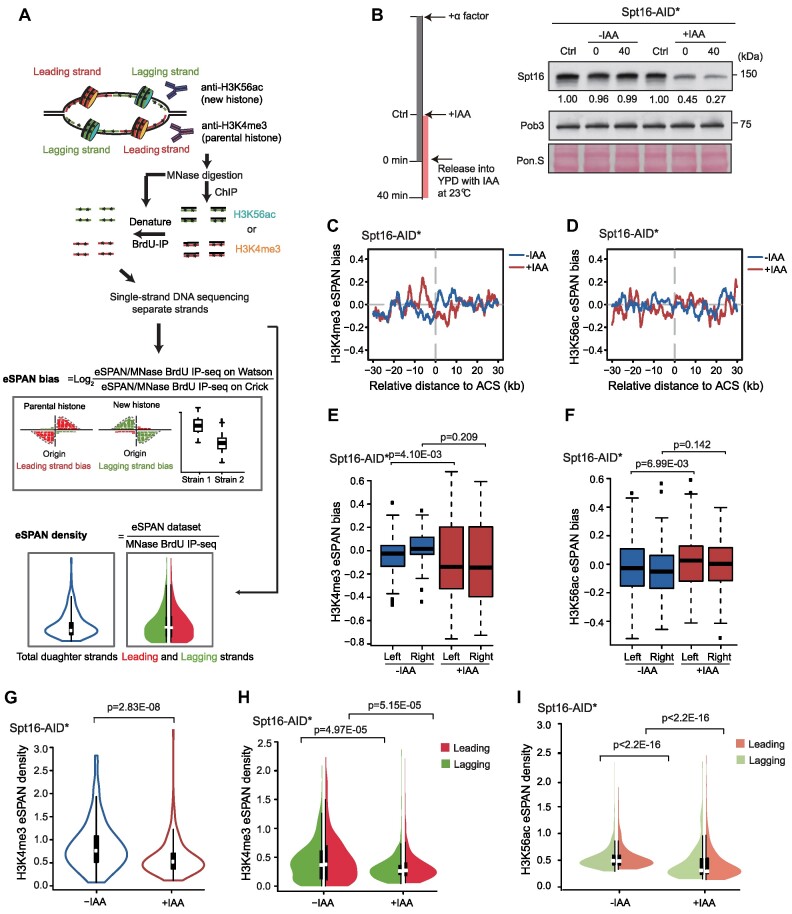
The FACT complex contributes to parental histone recycling on both the leading and lagging strands. (**A**) Graphical outline of the experimental procedure for eSPAN (enrichment and sequencing of protein-associated nascent DNA) analysis based on the hypothesis that parental and new H3/H4 are enriched at the leading and lagging strand, respectively. Briefly, yeast cells were synchronized at G1 phase and subsequently released into fresh YPD media containing 5-bromo-2-deoxyuridine (BrdU) for 40 min to label nascent DNA during DNA synthesis in early S phase. Chromatin in these cells was crosslinked with paraformaldehyde, digested using micrococcal nuclease (MNase) and then subjected to chromatin immunoprecipitation (ChIP) using antibodies against two different histone modifications: acetylation of lysine 56 of H3 (H3K56ac, a new histone mark) and tri-methylation of lysine 4 of H3 (H3K4me3, a parental histone mark). Subsequently, ChIP DNA was denatured into single-stranded DNA (ssDNA) and subjected to immunoprecipitation using an anti-BrdU antibody to enrich newly synthesized DNA. The resulting ssDNA was subjected to library construction and strand-specific deep sequencing. The MNase-digested chromatin was also denatured and subjected to BrdU IP as an input control. The eSPAN sequence reads mapped to the Watson and Crick strands were separated and normalized to the MNase-BrdU-IP-Seq dataset in order to minimize the difference in BrdU-incorporation. The average ratio of Watson/Crick sequence reads around early replication origins was calculated and termed eSPAN bias, which reflects the relative amount of a modified histone at the leading or lagging strand of DNA replication forks. The ratio of the total eSPAN sequence reads surrounding early replication origins to the total MNase-BrdU-IP-Seq reads in the same region was calculated and termed eSPAN density, which reflects the efficiency of parental histone recycling to nascent chromatin. The eSPAN density at the nascent leading and lagging strands was calculated separately. (**B**) Analysis of the protein level of Spt16 by immunoblotting using antibodies against the Spt16. Briefly, yeast cells were arrested in G1 phase by incubation with α-factor for 2.5 h; IAA was applied 1 h prior to the release to allow for Spt16 depletion. Cells were released into YPD media containing IAA at 23°C to allow entrance into early S phase, collected 40 min after release and subjected to immunoblotting and eSPAN analysis to evaluate the distribution of H3K4me3 and H3K56ac at replicating DNA forks. AID*: auxin-inducible degron. Pon. S: Ponceau S staining, as a loading control. (**C** and **D**) The average eSPAN bias of H3K4me3 (C) and H3K56ac (D) at early replication origins with (+IAA) or without (-IAA) Spt16 degradation. ACS: ARS (an autonomously replicating sequence) consensus sequence at replication origins. Similar results were obtained from at least two biological repeats; the second repeat is shown in Supplementary Figure S2. (**E** and **F**) The box plot of eSPAN bias of H3K4me3 (E) and H3K56ac (F) around early replication origins with (+IAA) or without (–IAA) Spt16 degradation. Left: the upstream of the origin (–30–0 kb); Right: downstream of the origin s(0–30 kb). Statistical significance was evaluated based on the Mann–Whitney *U* test. (**G**) The H3K4me3 eSPAN density at early replication origins with (+IAA) or without (–IAA) Spt16 degradation. Statistical significance was evaluated based on the Mann–Whitney *U* test. (**H** and **I**) The eSPAN density of H3K4me3 (H) and H3K56ac (I) on the leading (red) or lagging (green) strand at early replication origins with (+IAA) or without (–IAA) Spt16 degradation. Statistical significance was evaluated based on the Mann–Whitney *U* test.

The eSPAN sequence reads were separated and mapped the Watson and Crick strands, and the average Watson/Crick ratio of the eSPAN sequence reads around early replication origins was calculated and then normalized against the MNase-BrdU-IP-Seq dataset to minimize the BrdU-incorporation difference. This calculation, termed eSPAN bias, reflects the relative amount of a modified histone at the leading or lagging strands of DNA replication forks (Figure 1A). Considering that the eSPAN bias cannot detect the impact of a mutant on parental histone recycling if the mutant affects parental histone transfer to both the leading and lagging strands almost equally, we also calculated the ratio of total eSPAN sequence reads surrounding ACS to the total MNase-BrdU-IP-Seq reads in the same region in order to measure the efficiency of parental histone recycling on nascent strands, which we termed eSPAN density (Figure 1A). When eSPAN and BrdU-IP-Seq sequence reads were separated into Watson and Crick reads, the eSPAN density at nascent leading and lagging strand chromatin could also be calculated in this manner (Figure 1A).

In wild-type cells, the MNase-BrdU-IP-Seq signals around DNA replication origins were observed to span approximately 10 kb up- and downstream (Supplementary Figure S1A). Furthermore, a slight leading strand bias at early replication origins was detected in the MNase-BrdU-IP-Seq dataset, which is consistent with the notion that leading strand DNA synthesis proceeds further than that of the lagging strand (47) (Supplementary Figure S1B). A slight leading strand bias was also detected in both the H3K4me3 and H3K56ac eSPAN peaks in wild-type cells (Supplementary Figure S1B). Following normalization against MNase-BrdU-IP-Seq, neither the H3K4me3 (parental) nor the H3K56ac (new) eSPAN signals at early replication origins showed apparent bias for leading or lagging strands (Supplementary Figure S1C and D). Taken together, these data suggest that both parental and new H3/H4 are distributed almost equally onto the leading and lagging strands of DNA replication forks. In all following analysis, eSPAN signals normalized against BrdU-IP-ssSeq were used.

Since the two major subunits (Spt16 and Pob3) of FACT are essential in yeast cells, the auxin-inducible degron (AID*) system was employed to induce Spt16 degradation during early S phase (48). Auxin (Indole-3–acetic acid, IAA), a plant hormone that induces the degradation of AID*-tagged proteins, was added to the media at G1 phase 1 h prior to releasing the yeast cells into S phase, and protein levels were subsequently detected by immunoblotting (Figure 1B, left panel). The level of Spt16 was apparently decreased following IAA treatment and reduced to 27% during S phase but remained unchanged in the untreated control (Figure 1B, right panel). Without IAA, we observed a small but statistically significant leading strand bias for H3K4me3 eSPAN signals, but not H3K56ac eSPAN signals in in Spt16-AID* compared to wild type cells (Figure 1C–F, and Supplementary Figure S2A–I). This raises the possibility that the presence of the AID tag may compromise a function of FACT in parental histone transfer slightly towards the lagging strands. Upon Spt16 degradation, no noticeable bias for both H3K4me3 and H3K56ac eSPAN peaks at early replication origins was detected (Figure 1C–F and Supplementary Figure S2A–E), indicating that FACT depletion likely affects parental histone recycling or new histone deposition equally on both the leading and lagging strands, which could not be detected by eSPAN bias.

To explore this idea, we calculated the H3K4me3 and H3K56ac eSPAN density at early replication origins in untreated and Spt16-depleted cells (Figure 1G-I, Supplementary Figure S2J and K). In Spt16-depleted cells, the density of H3K4me3 eSPAN signals was reduced compared to untreated cells, suggesting impaired recycling of parental histones around early replication origins upon Spt16 degradation (Figure 1G and Supplementary Figure S2J). We also calculated the average H3K4me3 eSPAN density around these origins at both the leading and lagging strands, observed a similar decrease in both strands, with a slight statistical difference observed between the two strands in one of the two independent repeats (Figure 1H and Supplementary Figure S2K). Additionally, the average H3K56ac eSPAN density at both the leading and lagging strands also decreased upon Spt16 degradation (Figure 1I). Together, these results indicate that FACT plays a role in both the recycling of parental histone H3/H4 and the deposition of new H3/H4 equally at both the leading and lagging strands.

### The N-terminal domain of Spt16 contributes to parental histone transfer to the lagging strand

**Figure 2.**
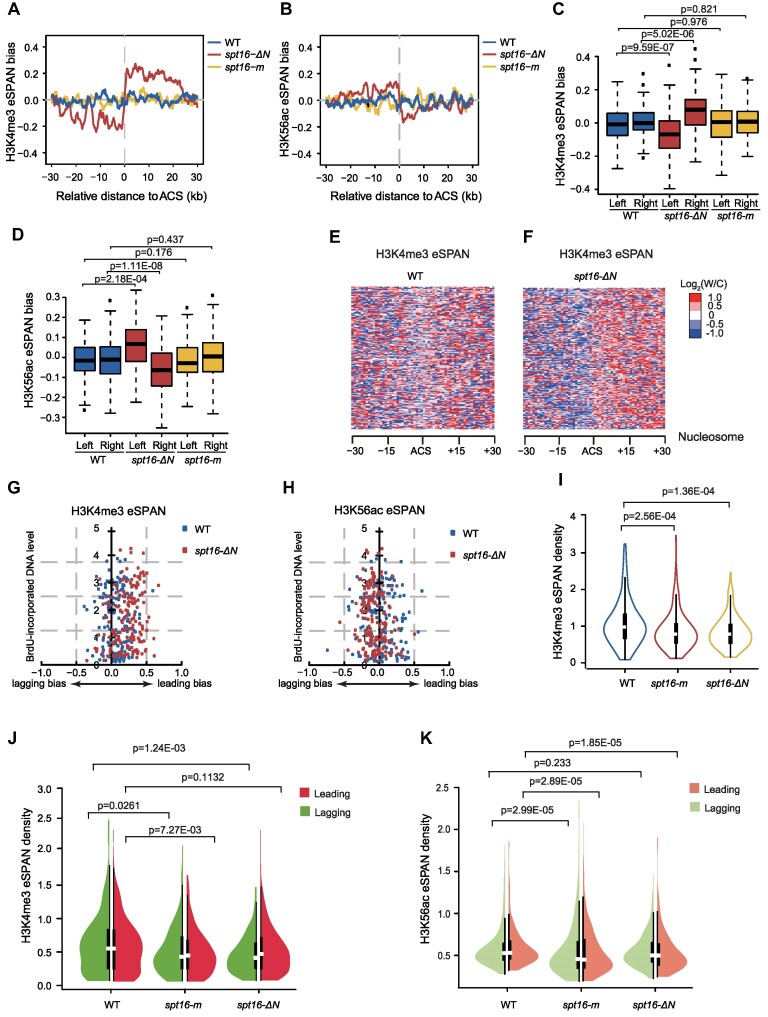
The Spt16-N domain contributes to parental histone recycling on the lagging strand. (**A** and **B**) The average eSPAN bias of H3K4me3 (A) and H3K56ac (B) at early replication origins in wild-type (WT), *spt16-m* and *spt16-ΔN* cells. (**C** and **D**) The box plot of H3K4me3 eSPAN bias around early replication origin in wild-type (WT), *spt16-m* and *spt16-ΔN* cells. Left: the upstream of the origin (–30–0 kb); Right: downstream of the origin (0–30 kb). Statistical significance was evaluated based on the Mann–Whitney *U* test. (**E** and **F**) Heatmap of H3K4me3 eSPAN peaks at each of the 30 individual nucleosomes around early DNA replication origins in wild-type (WT) and *spt16-ΔN* cells. (**G** and **H**) Correlation between the average eSPAN bias of H3K4me3 (G) or H3K56ac (H) and the BrdU-incorporation levels around early replication origins in wild-type (WT) and *spt16-ΔN* cells. (**I**) The H3K4me3 eSPAN density in wild-type (WT), *spt16-m* and *spt16-ΔN* cells at early replication origins. Statistical significance was evaluated based on the Mann–Whitney *U* test. (**J** and **K**) The eSPAN density of H3K4me3 (J) and H3K56ac (K) on the leading (red) or lagging (green) strand in wild-type (WT), *spt16-m* and *spt16-ΔN* cells at early replication origins. Statistical significance was evaluated based on the Mann–Whitney U test.

Recent studies, including our own have shown that the Spt16-N domain (*1–468 aa)* serves as a protein–protein interaction domain for factors involved in DNA replication, such as the MCM complex and the fork protection complex (33,34). Given the involvement of the Mcm2 histone binding domain (HBD) in parental histone transfer, we sought to further investigate FACT’s role in parental histone recycling and transfer by analyzing H3K4me3 eSPAN in the Spt16 mutant allele, s*pt16-ΔN* (deletion of amino acids 2–484 aa) (Figure 2A and Supplementary Figure S3A). Simultaneously, we performed H3K56ac eSPAN analysis in s*pt16-ΔN* mutant cells as a control (Figure 2B and Supplementary Figure S3A). Remarkably, we observed a mild but significant bias towards the leading strand for H3K4me3 eSPAN signals, as well as a corresponding bias towards the lagging strand for H3K56ac eSPAN signals in *spt16-ΔN* mutant cells (Figure 2A–E and Supplementary Figure S3B, C), suggesting that H3K4me3 signals are less enriched on the lagging strand than the leading strand upon deletion of the Spt16-N domain.

To rule out the biased distribution of H3K4me3 and H3K56ac at nascent chromatin in *spt16-ΔN* mutant cells being a result of defects in DNA replication in a strand specific manner, the correlation between H3K4me3 or H3K56ac eSPAN bias and the BrdU-incorporation level at each early replication origin was analyzed. Little correlation was found between H3K4me3 eSPAN bias and BrdU levels in either wild-type or *spt16-ΔN* mutant cells (Figure 2G and H). Moreover, the relative DNA synthesis rate at the leading and lagging strands, as measured by the MNase-BrdU-IP-Seq dataset, was similar in wild-type and *spt16-ΔN* mutant cells (Supplementary Figure S3D).

Analysis of the H3K4me3 eSPAN density in *spt16-ΔN* mutant cells revealed a reduction in recycled H3K4me3 signals compared to wild-type cells (Figure 2I). This reduction was particularly pronounced and statistically significant on the lagging strand, but not on leading strand (Figure 2J). In contrast, H3K56ac eSPAN density at leading, but not at lagging strands, in *spt16-ΔN* mutant cells was significantly reduced compared to wild-type cells, indicating a defect in new histone deposition specifically on the leading strand (Figure 2K and Supplementary Figure S3E). These findings collectively demonstrate the critical role of the Spt16-N domain in facilitating parental histone transfer to the lagging strands.

To confirm the unique role of the Spt16-N domain in parental histone dynamics, we analyzed the impact of the *spt16-m* mutant allele, which has previously been reported to exhibit defects in histone H3/H4 binding and H3K56ac deposition onto replicating DNA (36), on the distribution of parental and new H3/H4 at replication forks. Similar to Spt16 depletion, we detected no bias for H3K4me3 and H3K56ac eSPAN signals in *spt16-m* mutant cells (Figure 2A–D and Supplementary Figure S3A). As expected, the H3K56ac eSPAN density at both daughter strands was significantly reduced in the *spt16-m* mutant cells (Figure 2K and Supplementary Figure S3E). However, unexpectedly, we also observed a significant reduction of the H3K4me3 eSPAN density in both leading and lagging strands in *spt16-m* cells (Figure 2I and J). These results indicate that the Spt16-m mutation compromises both the deposition of new H3/H4 and the recycling of parental histone H3/H4, at both the leading and lagging strands to a similar extent.

Together, these results provide the first line of evidence that FACT functions in both the deposition of new H3/H4 and the recycling of parental H3/H4. Moreover, unlike Mcm2 (19,20), Dpb3 and Dpb4 (22), which mediate parental histone transfer to either the leading or lagging strand, FACT contributes to parental histone recycling on both the leading and lagging strands of the replicated regions. Furthermore, the Spt16-N domain plays a unique role in facilitating parental histone transfer to the lagging strands.

### The N-terminal domain of Spt16 contributes to the interaction of FACT with MCM

**Figure 3.**
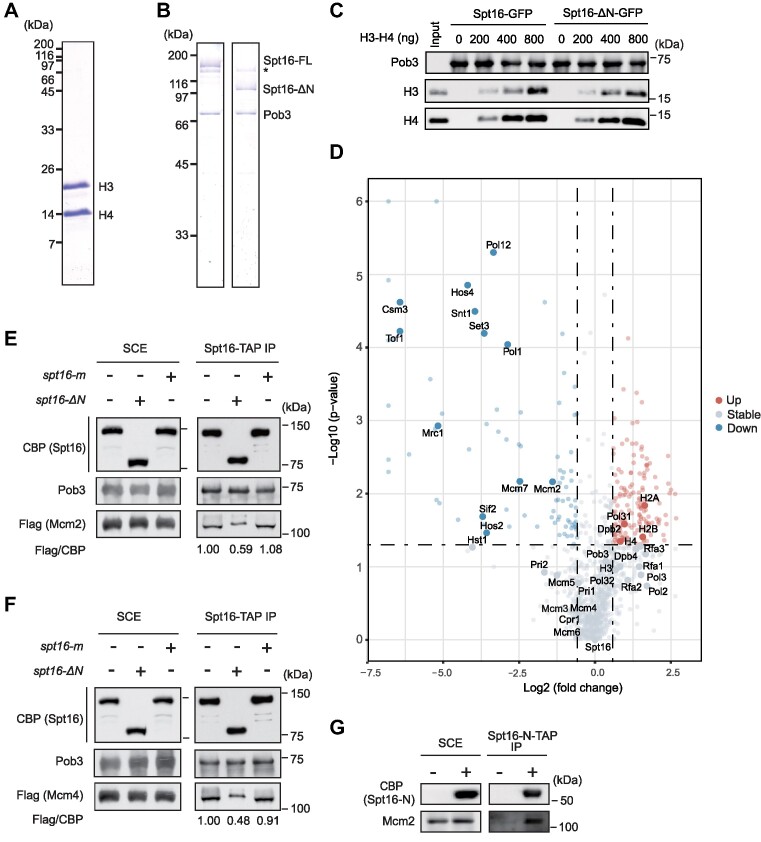
The Spt16-N domain is critical for the interaction between FACT and the MCM2–7 complex. (**A**) Recombinant yeast histone H3/H4 used in the *in vitro* pull-down assays. Recombinant yeast histone H3/H4 was purified from *E. coli* cells, resolved by SDS-PAGE and visualized by Coomassie Brilliant Blue (CBB) staining. (**B**) Yeast FACT complex containing GFP-tagged full-length (FL) or N-domain-deletion (ΔN) Spt16 used in the *in vitro* pull-down assays. GFP-tagged FL or ΔN Spt16 was purified from yeast cells, resolved by SDS-PAGE and visualized by CBB staining. *Non-specific proteins. (**C**) FACT complex lacking the Spt16-N domain does not impair the binding of H3/H4 with the FACT complex. FL and Spt16-ΔN FACT complexes were used to pull-down recombinant histone H3/H4, and the bound proteins were analyzed by immunoblotting using the indicated antibodies. At least three independent experiments were performed; one representative experiment is shown. (**D**) The amount of several replisome components is reduced in proteins co-purified with Spt16-ΔN. Wild-type (WT) Spt16 or Spt16-ΔN mutant proteins were purified from yeast cells using tandem affinity purification (TAP), and the associated protein complexes were resolved by SDS-PAGE and subjected to label-free quantitative mass spectrometry (MS). Representative proteins are visualized using volcano plots, and the corresponding data can be found in Supplementary Table S5. A comprehensive list of IP-MS results is provided in Tables S6 and S7. In the volcano plots, down-regulated associated proteins are depicted as blue dots, upregulated proteins are shown in red and proteins with no significant change are displayed in gray. The *P*-values were calculated using a multiple Student's *t*-test (*n* = 3). (**E**) The Mcm2 intensity associated with FACT is reduced in the *spt16-ΔN* cells. TAP-tagged Spt16 was purified from WT, *spt16-ΔN* and *spt16-m* yeast cells expressing Flag-tagged Mcm2. The co-purified proteins (IP) were resolved by SDS-PAGE and detected using the indicated antibodies. CBP, calmodulin-binding peptide; SCE, soluble cell extracts. (**F**) The Mcm4 intensity associated with FACT is reduced following the Spt16-N domain deletion. The experiments were performed as described in (E). (**G**) The Spt16-N domain interacts with Mcm2 in cells. The TAP-tagged Spt16 N-terminal domain (Spt16-N-TAP) was expressed (+) and purified from yeast cells. Co-purified proteins were separated by SDS-PAGE and detected using specific antibodies. CBP, calmodulin-binding peptide; SCE, soluble cell extracts. Cells without Spt16-N-TAP expression served as a negative control.

It has been reported that the N-terminus of Spt16 is capable of binding to histone H3/H4 *in vitro* (39), raising the possibility that the impaired parental histone transfer in the *spt16-ΔN* mutant cells may be attributed to compromised histone binding. To test this hypothesis, we performed an *in vitro* pull-down assay using FACT complex purified from wild-type or *spt16-ΔN* mutant yeast cells, along with recombinant yeast histone H3/H4 purified from *E. coli* cells (Figure 3A and B). Interestingly, we observed no apparent reduction in the association between H3/H4 and mutant FACT lacking the Spt16 N-terminus, as compared to the wild-type FACT complex (Figure 3C). To further confirm this observation, an *in vivo* tandem affinity purification (TAP) assay was performed using TAP-tagged Spt16 with or without its N-terminus. Consistent with the *in vitro* pull-down assay, the amount of co-purified histones with FACT was not decreased in *spt16-ΔN* mutant cells compared to wild-type cells (Supplementary Figure S4A). These results suggest that other domains within the FACT complex are responsible for mediating the interaction between FACT and histones, and that the N-terminus of Spt16 contributes to parental histone recycling through alternative mechanism(s).

Next, we investigated whether the Spt16-N domain is critical for the FACT–MCM2–7 interaction by affinity purification–mass spectrometry (AP–MS). Mass spectrometry (MS) revealed that all the subunits of the MCM complex co-purified with Spt16-TAP (Figure 3D and Supplementary Table S5). Moreover, other replisome components, including the fork protection complex (Mrc1, Tof1, Csm3), the Pol α, the Pol ϵ and the Pol δ complexes, were also detected. As expected, the Set3C complex, which consists of Set3, Sif2, Hos4, Snt1, was also co-purified with Spt16. Consistent with previous finding that co-purified Set3C complex subunits were markedly reduced in the *spt16-ΔN* mutant compared to wild type Spt16 (34). Interestingly, the abundance of several co-purified replisome components, including the MCM2–7 complex, Tof1/Mrc1/Csm3 and the DNA polymerase α complex, was significantly decreased in *spt16-ΔN* mutant cells compared to wild-type cells. In contrast, the abundance of several subunits of the Pol ϵ and Pol δ complexes co-purified with the Spt16-ΔN appeared to be similar to that of wild-type Spt16. These results suggest that the Spt16 N domain contributes to the interaction between FACT and specific replisome components, including the Mcm2–7 complex, the Mrc1-Tof1-Csm3 complex and the DNA Pol α complex.

It has been shown that histone H3/H4 proteins bridge the interaction between the Mcm2HBD and FACT (28). Mutations in the Mcm2HBD (*mcm2–3A*, *Y79A, Y82A, Y91A*), which disrupt the interaction between Mcm2HBD and histone H3/H4 and the transfer of parental histones to lagging strands, also prevent the interaction between FACT and Mcm2HBD (28,49). Given that both the deletion of the Spt16-N domain and mutations of the Mcm2HBD result in defects in parental histone transfer to the lagging strand (Figure 2) (22), we focused on investigating the impact of Spt16-ΔN on the FACT–MCM interaction (Figure 3E–G, and Supplementary Figure S4B). Consistent with the MS results, immunoblotting analysis demonstrated a reduction in the amount of Mcm2 and Mcm4 co-purified with Spt16-*Δ*N-TAP compared to wild-type Spt16-TAP (Figure 3E and F). In contrast, the levels of Mcm2 co-purified with Spt16-m proteins were a slightly more than that co-purified with wild-type Spt16 (Figure 3E), and the amount of Mcm4 co-purified with Spt16-m proteins was similar to those co-purified with wild-type Spt16 (Figure 3F). Together, these results indicate that the Spt16-N domain has a unique role in mediating the interaction between FACT and the MCM2–7 complex in cells.

Spt16 N-domain alone lacks the ability to form a complex with Pob3 and cannot interact with H3/H4 in cells (34). We therefore overexpressed the TAP-tagged Spt16-N domain alone in cells and tested whether this domain, expressed alone, could interact with Mcm2–7 complex *in vivo*. We observed Mcm2 co-purified with the Spt16 N domain (Figure 3G). These results strongly suggest that the Spt16 N-terminus directly interacts with the MCM2–7 complex in cells.

To investigate whether the ability of Mcm2 to bind H3/H4 is needed for FACT to interact with MCM2–7 complex, we purified the Spt16-TAP complex from both wild-type and *mcm2–3A* mutant cells. Surprisingly, we did not observe a significant reduction in the binding of Mcm2–3A mutant proteins to the Spt16 complex compared to wild-type Mcm2 (Supplementary Figure S4B). Collectively, these results indicate that, while H3/H4 brides the interaction between Mcm2HBD and FACT, FACT likely interacts with MCM2–7 complex mainly through other means. Because deletion of Spt16 N-terminus reduces FACT-MCM2–7 interaction, Spt16 N-domain likely interacts with MCM2–7 through regions other than Mcm2HBD domain.

### The N-terminus of Spt16 does not affect FACT or MCM2–7 binding at chromatin surrounding early replication origins

Both FACT and MCM2–7 travel along with replication forks. Therefore, we assessed whether the Spt16 N-terminus is important for FACT or MCM2–7 to bind chromatin at G1 and S phase. Briefly, chromatin immunoprecipitation (ChIP) of Spt16 and Mcm6 was performed in wild-type and *spt16-ΔN* mutant cells at G1 or S phase. The N-terminal deletion of Spt16 did not impair the chromatin binding of FACT in G1 or early S phase (Supplementary Figure S5A–D). Moreover, N-terminal deletion of Spt16 had little effect on Mcm6 binding at replication origins, *ARS607* and *ARS305*, in G1 phase or during S phase progression (Supplementary Figure S5E and F). The distribution of PCNA (proliferating cell nuclear antigen), which serves as a clamp for both DNA polymerases ϵ and δ, on replicating DNA was also not affected in *spt16-ΔN* cells in comparison with wild-type cells (Supplementary Figure S5G and H). Consistent with these results, DNA content analysis by FACS revealed no apparent defects on the progression of S phase in *spt16-ΔN* cells compared to wild-type cells (Supplementary Figure S6A and B). Thus, deletion of the Spt16-N domain has little impact on the chromatin binding of FACT, Mcm2–7 and PCNA. These results suggest that the Spt16-N domain likely mediates the interaction of FACT with MCM2–7 for parental histone transfer during DNA replication.

### The N-terminus of Spt16 and Mcm2HBD play a crucial role in parental histone recycling

**Figure 4.**
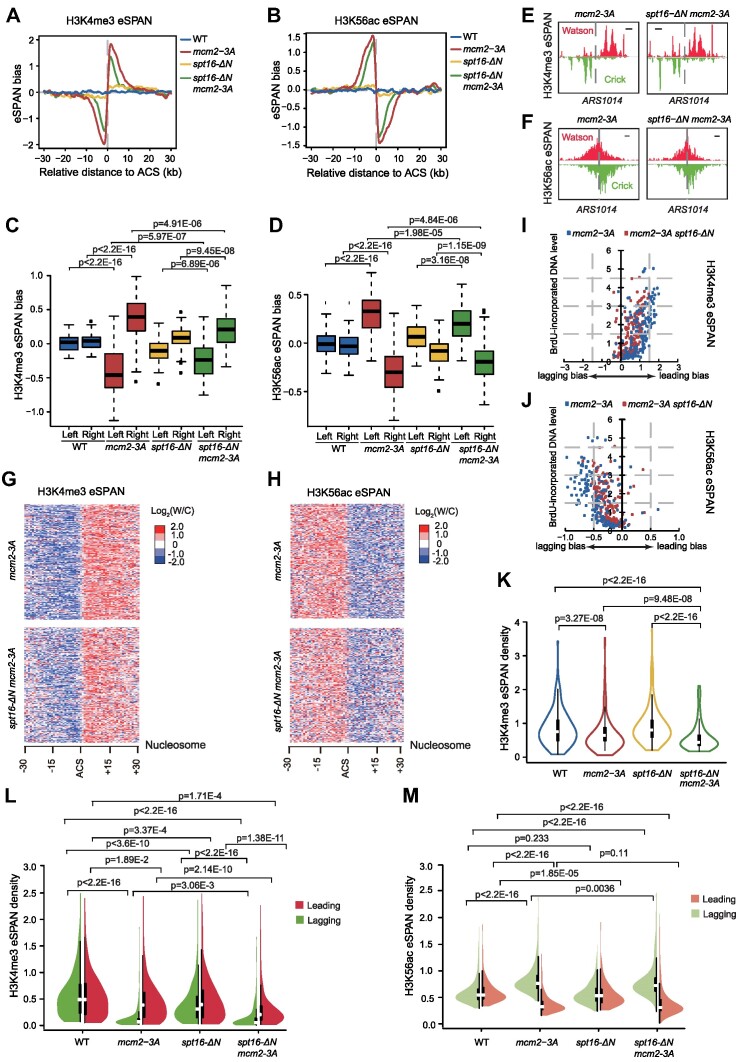
Effects of *spt16-ΔN* mutation on parental histone transfer when combined with the *mcm2–3A* mutant. (**A** and **B**) The average eSPAN bias of H3K4me3 (A) and H3K56ac (B) at early replication origins in wild-type (WT), *mcm2–3A*, *spt16-ΔN* and *mcm2–3A spt16-ΔN* cells. Similar results were obtained from at least two biological repeats; the second repeat is shown in Supplementary Figure S7. (**C** and **D**) The box plot of H3K4me3 (C) and H3K56ac (D) eSPAN signal around early replication origin in wild-type (WT), *mcm2–3A*, *spt16-ΔN* and *mcm2–3A spt16-ΔN* cells. Statistical significance was evaluated based on the Mann–Whitney *U* test. (**E** and **F**) Snapshot of H3K4me3 (E) and H3K56ac (F) eSPAN peaks at the region surrounding replication origin *ARS1014* in *mcm2–3A* and *mcm2–3A spt16-ΔN* cells. The scale bar represents a 2 kb DNA region. (**G** and **H**) Heatmap of H3K4me3 (E) and H3K56ac (F) eSPAN peaks at each of the 30 individual nucleosomes around early DNA replication origins in *mcm2–3A* and *mcm2–3A spt16-ΔN* cells. (**I** and **J**) Correlation between the extent of the average eSPAN bias of H3K4me3 (I) or H3K56ac (J) and the BrdU-incorporation level around early replication origins in *mcm2–3A* and *mcm2–3A spt16-ΔN* cells. (**K**) The average H3K4me3 eSPAN density at early replication origins in WT, *mcm2–3A*, *spt16-ΔN* and *mcm2–3A spt16-ΔN* cells. Statistical significance was evaluated based on the Mann–Whitney U test. (**L** and **M)** The average H3K4me3 (L) and H3K56ac (M) eSPAN density at the leading (red) or lagging (green) strand in WT, *mcm2–3A*, *spt16-ΔN* and *mcm2–3A spt16-ΔN* cells at early replication origins. Statistical significance was evaluated based on the Mann–Whitney *U* test.

Similar to *spt16-ΔN* mutant cells, *mcm2–3A* mutant cells also show defects in parental histone transfer to the lagging strand in budding yeast, albeit more pronounced than *spt16-ΔN* cells (20,22). Therefore, we evaluated the relationship between the Spt16-N domain and the Mcm2HBD in the process of parental histone transfer. For this purpose, we first generated a *spt16-ΔN mcm2–3A* double-mutant yeast strain and used the eSPAN method to evaluate the impact of double mutations on parental histone transfer. In comparison with the *mcm2–3A* single mutant, we observed a mild yet statistically significant reduction in the extent of the bias towards the leading and lagging strand for H3K4me3 eSPAN peaks (Figure 4A, C, E, G and Supplementary Figure S7A and C) and for H3K56ac eSPAN peaks (Figure 4B, D, F, H and Supplementary Figure S7B and D), respectively, in *spt16-ΔN mcm2–3A* double-mutant cells. The correlation between H3K4me3 or H3K56ac eSPAN bias and the BrdU-incorporation level at each fired replication origin was calculated, revealing no correlation between the extent of bias reduction in *spt16-ΔN mcm2–3A* double-mutant cells and the BrdU-incorporation level (Figure 4I and J). The reduction of the extent of H3K4me3 eSPAN bias towards the leading strand in *mcm2–3A spt16-ΔN* double mutant cells could be due to either an increase in H3K4me3 at lagging strand or a decrease in H3K4me3 levels on the leading strand compared to those observed in the *mcm2–3A* mutant alone.

To distinguish between these two possibilities, we analyzed the eSPAN density of the regions 2 kb up- and downstream of the early replication origins in *spt16-ΔN* and *mcm2–3A* single*-*mutant and *spt16-ΔN mcm2–3A* double-mutant cells. We observed a significant reduction in the average H3K4me3 eSPAN density in *mcm2–3A* cells, which was further reduced in *spt16-ΔN mcm2–3A* double-mutant cells (Figure 4K and Supplementary Figure S7E, F). To further evaluate parental histone transfer to the leading or lagging strand, we calculated the H3K4me3 eSPAN density separately for each strand (Figure 4L). In both *mcm2–3A* and *spt16-ΔN* single-mutant cells, the H3K4me3 eSPAN density was significantly reduced at both leading and lagging strand compared to wild type. Furthermore, the reduction of H3K4me3 eSPAN density at lagging strand was more much pronounced than leading strand (Figure 4L), consistent with the findings from the eSPAN bias analysis of a preferential reduction in parental histone transfer to the lagging strand in these two single mutant strains. Notably, the eSPAN density on the lagging strand was further reduced in s*pt16-ΔN mcm2–3A* double-mutant cells (Figure 4L), indicating that both FACT and Mcm2 are critical for efficient parental histone transfer to the lagging strand. Surprisingly, the eSPAN density on the leading strand in s*pt16-ΔN mcm2–3A* double-mutant cells was also significantly reduced compared to that in *mcm2–3A* or *spt16-ΔN* single-mutant cells (Figure 4L). These results suggest that in the absence of the Spt16 N-domain, the transfer of parental histones to the leading strand in *mcm2–3A* mutant background is also impaired, providing an explanation for the reduced H3K4me3 eSPAN strand bias in s*pt16-ΔN mcm2–3A* double-mutant cells compared to the single-mutant cells. These results provide additional support to the idea that FACT has a role in parental histone transfer, and it does so in pathways in both Mcm2HBD dependent and independent manners.

We also examined the H3K56ac eSPAN density on each strand to assess the deposition of new histones on the leading or lagging strand (Figure 4M). We observed a significant reduction in the H3K56ac eSPAN density on the leading strand in *mcm2–3A* single-mutant cells (Figure 4M). In contrast, the lagging strand of *mcm2–3A* and *spt16-ΔN* cells mutant cells showed an elevated H3K56ac eSPAN density. This increase in H3K56ac eSPAN density on the lagging strand is likely due to impaired transfer of parental histones to lagging strands, and thereby increasing demand for new histones to fill in the gaps left by parental histones. Interestingly, *spt16*-*ΔN* had a weak but significant impact on the H3K56ac eSPAN density at the lagging strand in the *mcm2–3A* background. These findings collectively suggest that the interaction between FACT and Mcm2, mediated by the Spt16-N domain, plays a role in parental histone recycling and also influences H3K56ac deposition dynamics.

### The N-terminus of Spt16 regulates the formation of a ternary complex comprising FACT, histones and Mcm2HBD *in vitro*

**Figure 5.**
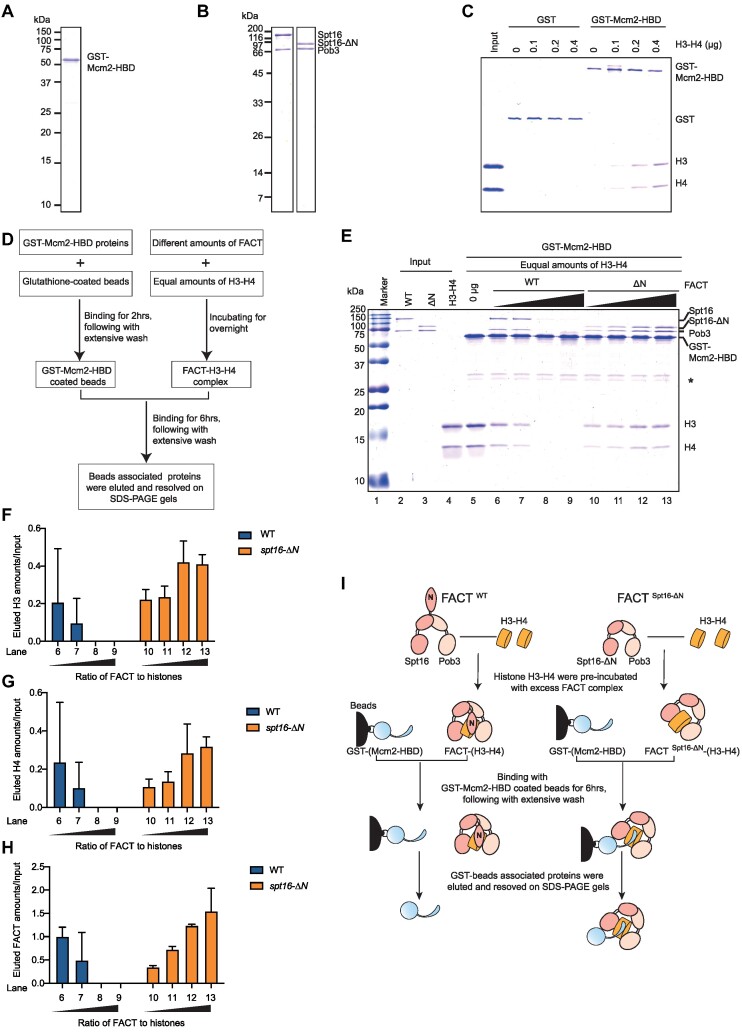
The Spt16-N domain protects FACT-bound histone H3/H4. (**A**) The recombinant yeast GST-tagged Mcm2HBD (residues 1–200) was used in the *in vitro* histone capture assay. The recombinant GST-Mcm2HBD was purified from *E. coli* cells, resolved by SDS-PAGE and visualized by Coomassie Brilliant Blue (CBB) staining. (**B**) FACT containing TAP-tagged full-length Spt16 or Spt16-ΔN used in the *in vitro* histone capture assay. TAP-tagged full-length Spt16 or Spt16-ΔN protein complexes were purified from yeast cells, resolved by SDS-PAGE and visualized by CBB staining. (**C**) The recombinant GST-Mcm2HBD binds to histone H3/H4. Equal amounts of GST or the GST-Mcm2HBD was bound to GST beads and subjected to an *in vitro* pull-down assay. An increasing amount of histone H3/H4 was added to each reaction, and the bound proteins were resolved by SDS-PAGE and visualized by CBB staining. (**D**) Graphical outline of the experimental procedure for the *in vitro* histone capture assay. Briefly, equal amounts of histones were pre-incubated with increasing amounts of either the wild-type (WT) or Spt16ΔN form of the FACT complex overnight, promoting the formation of the FACT–histone complex. The resulting reaction products were then subjected to a pull-down assay using GST-Mcm2HBD coated beads. After 6 h of rotation binding, the beads were extensively washed and the GST-Mcm2HBD -associated complex was eluted and resolved on SDS-PAGE gels. (**E**) The Spt16-N domain prevents the capture of FACT-bound histone H3/H4 by the GST-Mcm2HBD proteins. Increasing amounts of WT and Spt16-ΔN FACT complex were incubated overnight with 400 ng recombinant histone H3/H4 to allow for the formation of the FACT–H3/H4 complex. These proteins were then mixed with an equal amount of GST-Mcm2HBD-coated beads and subjected to an *in vitro* pull-down assay. The recovered protein complex was resolved by SDS-PAGE and visualized by CBB staining. *Non-specific proteins. Similar results were obtained from at least three independent experiments; one representative experiment is shown. (**F** and **G**) Quantitation of histone H3 (F) and H4 (G) captured by the GST-Mcm2HBD in (E). The intensity of histone H3 and H4 bands in (E) was quantitated using ImageJ and normalized to the input of the same lane. The quantitated results are from three independent experiments. (**H**) Quantitation of FACT pulled down by the GST-Mcm2HBD in (E). The intensity of Pob3 bands in (E) were quantitated using Image J and normalized to the input of the same lane. The quantitated results are from three independent experiments. (**I**) A proposed model illustrating the events of histone capture from the wild-type (WT) or Spt16-ΔN FACT-bound histone complex can be outlined. In the WT FACT–H3/H4 complex, the Spt16-N domain collaborates with other H3/H4-binding domains within the FACT complex to safeguard histone H3/H4. This protective mechanism prevents the easy exposure or leakage of histone H3/H4. Consequently, the GST-Mcm2HBD encounters difficulty in accessing histone H3/H4 when it is bound to the intact FACT complex. In contrast, within the Spt16-ΔN-containing complex, the FACT-bound histone H3/H4 is more readily exposed or accessed. This increased accessibility allows for its capture by the GST-Mcm2HBD.

To gain further insight into the connection between the Spt16-N domain and the Mcm2HBD during parental histone recycling and transfer, we investigated whether FACT cooperates with Mcm2HBD for histone binding and further whether Spt16 N-domain has a role in this process. We purified recombinant GST-tagged Mcm2HBD (GST-Mcm2HBD) from *E. coli* cells (Figure 5A) and confirmed that it bound to yeast histone H3/H4 through a GST pull down assay (Figure 5C). We also purified wild-type FACT (FACT WT) and a mutant FACT lacking the Spt16-N domain complex (FACTΔN) from yeast cells under stringent conditions (Figure 5B). We assessed the interaction between FACT and the GST-Mcm2HBD proteins using a GST-pull-down assay, and observed no detectable interaction between FACT and the GST-Mcm2HBD *in vitro* (Supplementary Figure S8A). Preincubation of increasing amounts of recombinant histone H3/H4 with the GST-Mcm2HBD resulted in the pull-down of increasing amounts of histone H3/H4 (Figure 5C). When an equal amount of FACT was added to the reaction, the pull-downed FACT was also increased along with the increased amounts of histones (Supplementary Figure S8C), consistent with previous studies demonstrating that histone H3/H4 mediates the interaction between FACT and the Mcm2HBD in cells (28). Interestingly, deletion of the Spt16-N domain did not affect the binding of FACT to the Mcm2HBD–H3/H4 complex (Supplementary Figure S8B and C), suggesting that the Spt16-N domain does not directly participate in the formation of the GST-Mcm2HBD–H3/H4–FACT complex. This result is also consistent with the observation that depletion of the Spt16 N-terminus did not impact the binding of FACT to H3/H4 *in vitro* or *in vivo*.

Next, we asked whether the ternary complex of FACT–H3/H4–Mcm2HBD can also be formed efficiently when FACT binds H3/H4 first (Figure 5D–H). Considering that GST-Mcm2HBD does not bind FACT *in vitro*, the capture of histones by GST-Mcm2HBD proteins can only occur when the GST-Mcm2HBD–H3/H4–FACT ternary complex is formed. We performed a GST-pull down assay using GST-Mcm2HBD after equal amounts of histones were pre-incubated with increasing amounts of either wild-type (WT) or Spt16ΔN form of the FACT complex to promote the formation of the FACT–(H3/H4) complex first. Surprisingly, as the amount of FACT increased during the formation of the FACT–H3/H4 complex, we observed a decrease in the levels of both histones and FACT pulled down by GST-Mcm2HBD beads (Figure 5E–H, lanes 6–9). This decrease is likely due to the binding of all histones in the solution to the FACT WT complex, protecting them from exposure or accessibility by GST-Mcm2HBD proteins on beads. Surprisingly, at the same molar ratio of FACT to histones, we observed an increase in the levels of histones and FACTΔN pulled down by GST-Mcm2HBD beads (Figure 5E–H, lanes 10–13). This increase is unlikely due to non-specific binding, as we did not observe retention of the FACTΔN–H3/H4 complex by GST-coated beads alone (Supplementary Figure S8D and E). These results suggest that in the absence of the Spt16-N domain, H3/H4 can be more readily exposed or accessed by GST-Mcm2HBD, leading to the formation of the Spt16-ΔN FACT–H3/H4–Mcm2HBD complex *in vitro* (Figure 5I). Thus, our results suggest that the Spt16-N domain likely also plays a role in regulating the exposure or accessibility of FACT-bound histones to Mcm2HBD, thereby influencing the coordination between FACT and Mcm2 in parental histone recycling and transfer.

### The Spt16 N-terminus regulates the interaction between Mcm2 and histone H3/H4 *in vivo*

**Figure 6.**
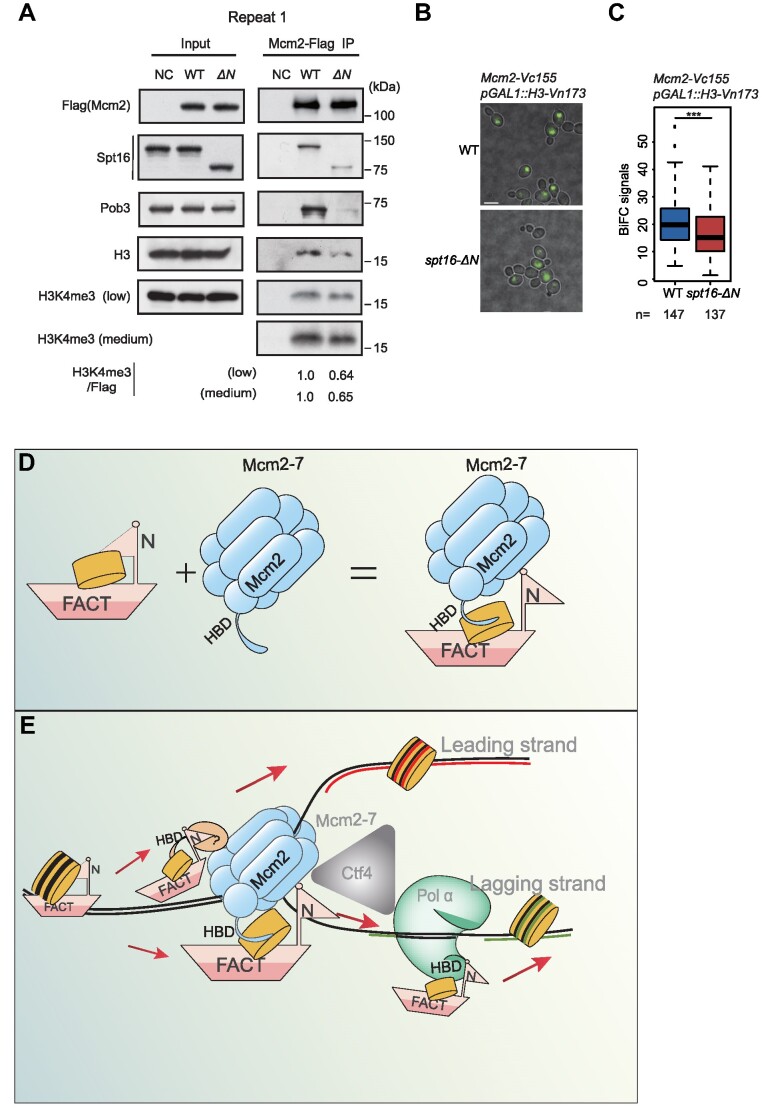
The Spt16-N domain anchors FACT to the replisome for parental histone recycling. (**A**) The Spt16-N domain is important for the binding of Mcm2 to histone H3 in cells. Flag-tagged Mcm2 was purified from wild-type (WT) and *spt16-ΔN* (ΔN) yeast cells, a strain without flag as control (NC). The co-purified proteins (IP) were resolved by SDS-PAGE and detected using the indicated antibodies. Similar results were obtained from at least four independent experiments; one representative experiment is shown. ‘Low’ and ‘Medium’ refer to the exposures of the H3K4me3 bands by western blot analysis. The intensity of H3K4me3 and Mcm2-Flag bands were quantitated and the ratio of H3K4me3/Flag was calculated. (**B**) BiFC intensity of *Mcm2-Vc155 pGAL1-H3-Vn173* cells with or without the *spt16-ΔN* mutant. Histone H3 was induced with 2% galactose for 2.5 h prior to imaging. The scale bar represents 5 μm. Similar results were obtained from at least three independent experiments; one representative experiment is shown. (**C**) Quantitation of the fluorescence intensities in (B). Statistical significance was evaluated based on *t*‐tests (***P < 0.001). (**D** and **E**) A working model is proposed to illustrate the role of the Spt16-N domain in regulating the formation of the ternary complex composed of FACT, histones and the Mcm2–7 complex. In this model, the Spt16-N domain serves two key functions: Firstly, it safeguards the integrity of FACT-bound histones, preventing their exposure or release. Secondly, it acts as an anchor, facilitating the interaction between FACT and the MCM2–7 helicase. Once this interaction is established, the FACT-bound histone H3/H4 is exposed for interaction with the Mcm2HBD, facilitating the parental histone recycling to lagging strands (D). In addition to Mcm2HBD, our experimental results also suggest that FACT likely also collaborates with other replisome components for the transfer of parental histones at the replicating regions (E). HBD: histone binding domain.

Due to the important role of the Spt16-N domain in the interaction between FACT and the Mcm2–7 complex in a manner independent of the Mcm2HBD, we analyzed how deletion of the Spt16-N domain affects the formation of FACT–H3/H4–MCM complex in cells. A Flag tag was engineered at the C-terminus of Mcm2 and used to purify Mcm2-associated proteins from wild-type and *spt16-ΔN* yeast cells (28). We found that both histone H3 and Spt16 were co-purified with Mcm2-Flag (Figure 6A and Supplementary Figure S9A–C). Remarkably, the amount of co-purified histone H3 and Spt16 were decreased in *spt16-ΔN* mutant compared to wild-type (Figure 6A and Supplementary Figure S9A–C). Additionally, the presence of parental histones marked by H3K4me3 was also reduced in *spt16-ΔN* mutant cells (Figure 6A and Supplementary Figure S9A–C). We barely detected H3K56ac signals under these conditions, indicating that the majority of histones associated with Mcm2-Flag were released from parental chromatin. These results support the notion that the Spt16 N-terminus domain is critical for the formation of FACT–H3/H4–Mcm2 complexes in cells. In the absence of the Spt16 N-terminus, the interactions between FACT and MCM are diminished, resulting in reduced amount of ternary complex (see Discussion).

To further test this idea, we employed a bimolecular fluorescence complementation (BiFC) assay to evaluate the interaction between Mcm2 and histones in live cells with or without the Spt16-N domain. The C-terminus of yellow fluorescent protein (YFP), Vc155, was fused to endogenous Mcm2, and the N-terminus of YFP (Vn173) was fused to histone H3 under the control of a *GAL1* promoter on the pRS426 plasmid. Following induction with galactose, exogenous histone H3-Vn173 was expressed in both wild-type and *spt16-ΔN* mutant cells that also expressed Mcm2-Vc155. The expression level of histone H3 was comparable between in wild-type and *spt16-ΔN* mutant cells (Supplementary Figure S10A). The split YFP would reconstitute and become detectable if an interaction occurred in the cells. Indeed, YFP fluorescence was observed in cells co-expressing the Mcm2-Vc155 and H3-Vn173 constructs (Figure 6B). Importantly, YFP intensity of YFP fluorescence was significantly reduced in *spt16-ΔN* mutant cells (Figure 6B and C). As a control, we analyzed the interaction between Mcm2-Vc155 and Mcm5-Vn173 using the same BiFC assay in wild-type and *spt16-ΔN* mutant cells, and found that the interaction between these two proteins was not affected by the deletion of the Spt16-N domain (Supplementary Figure S10B and C). These results collectively support the idea that the Spt16 N-terminus regulates the interaction between FACT and Mcm2–7, thereby influencing the cooperation between FACT and Mcm2 HBD in the transfer of parental histone H3/H4.

## Discussion

In this study, we revealed the involvement of the histone chaperone FACT in parental histone recycling and transfer during DNA replication. Our findings demonstrate that besides its role in new histone deposition, FACT is essential for the efficient recycling and transfer parental histone to both the leading and lagging strands of DNA replication forks. Surprisingly, we have also identified a previously unknown role for the Spt16 N-terminal domain in facilitating parental histone transfer. This domain interacts with the MCM replicative helicase, enabling the formation of a ternary complex comprising of FACT, H3/H4 histones and Mcm2HBD. Below, we discuss the implication of these findings with a focus on the role of FACT in parental histone transfer.

### FACT regulates parental histone recycling to both the leading and lagging strands of DNA replication forks

To facilitate access of the DNA replication machinery to nucleosomal DNA, nucleosomes containing parental histones with specific modifications must be disassembled ahead of DNA replication forks and subsequently recycled and transferred onto nascent strands. This process of parental histone recycling and transfer is critical for the inheritance of epigenetic information. While its importance is well recognized, the underlying mechanisms remain poorly understood. Early studies proposed that parental histones are randomly and evenly distributed onto newly synthesized strands (50,51). However, experiments conducted in *Xenopus* cell extracts and the SV40 DNA replication system have revealed contrasting results. In *Xenopus* cell extracts, parental histone H3/H4 can be locally recycled, suggesting a more specific transfer mechanism. In contrast, in the SV40 system, parental histone H3/H4 is dispersed without any discernible pattern (52). The major difference between these two replication systems is the involvement of different replicative DNA helicases. In *Xenopus*, the MCM2–7 helicase complex is responsible, whereas the SV40 replication system employs Large T antigen being the helicase. More recently, studies employing biotin-based labeling system have shed further light on parental histone dynamics. By labeling histone H3 in parental nucleosomes at specific loci, it has been demonstrated that parental nucleosomes largely retain their original positions after DNA replication (12,13). These findings support the active involvement of replisome components in the process of parental histone transfer. Indeed, Mcm2, a subunit of the Mcm2–7 complex, binds to released parental H3/H4 and regulates its transfer to the lagging strand through the Mcm2–Ctf4–Polα axis (20,22,28). Additionally, Dpb3 and Dpb4, subunits of the DNA polymerase ϵ complex, bind to histone H3/H4 and contribute to parental histone transfer to the leading strand (19). These studies highlight the complex and coordinated nature of parental histone recycling and transfer during DNA replication.

In addition to these replisome components, the histone chaperone FACT has been proposed to play a role in parental histone transfer. For instance, it has been shown that FACT interacts with key replisome components, such as Mcm2, and cooperatively binds to chromatin released histones (28). Additionally, FACT has been demonstrated to be essential for chromatin replication in the *in vitro* reconstituted replication system using chromatin templates (16). However, the *in vivo* evidence supporting the involvement of FACT in parental histone transfer has been limited. Furthermore, it remains unknown whether FACT operates similarly to Mcm2-Ctf4-Polα and Dpb3-Dpb4 in mediating the transfer of parental histones to either the leading or lagging strand during DNA replication.

Here, we provide compelling *in vivo* evidence supporting the involvement of FACT in parental histone transfer to both the leading and lagging strands of DNA replication forks. Depletion of Spt16, an essential subunit of the FACT complex, or expression of a histone binding-defective *spt16-m* mutant, leads to reduced levels of H3K4me3 at both the leading and lagging strands, of nascent chromatin, as measured by eSPAN. Notably, in contrast to the specific roles of Mcm2, Dpb3/POLE3 and Dpb4/POLE4 in regulating parental histone transfer to one strand of the DNA replication fork, the FACT complex appears to have a broader influence, operating at both strands.

Furthermore, our findings reveal a notable reduction in parental histone H3K4me3 recycling in yeast cells lacking the Spt16-N domain, particularly at the lagging strand compared to the leading strand. This finding further supports FACT’s involvement in parental histone transfer. These collective findings present compelling *in vivo* evidence supporting the essential role of FACT in the intricate process of parental histone transfer. The distinct impact of the Spt16-N domain on parental histone recycling at the leading and lagging strands emphasizes the complex and multifaceted involvement of the FACT complex in this critical biological process.

### A mechanism of action for the Spt16 N domain in parental histone transfer

To understand the manner by which the Spt16 N-terminal domain functions in parental histone transfer, we explored several possibilities. Initially, we investigated whether the depletion of the Spt16-N domain (residues 2–484) affects histone binding, as previous *in vitro* studies suggested its interaction with the H3/H4 dimer. Surprisingly, we found that the FACT complex lacking the N-terminal domain maintained its ability to interact with histone H3/H4 both *in vitro* and in cells. Recent studies on the structure of the human Spt16-N domain revealed that a long disordered region called hSpt16-LDR binds to H3/H4 tetramers *in vitro* (53). Interestingly, the region in yeast Spt16 (ySpt16) corresponding to hSpt16-LDR spans residues 458–534 and remains largely intact in *spt16-ΔN* mutant cells. This observation provides a potential explanation for the minimal impact of the Spt16-ΔN mutant on H3/H4 binding. Additionally, besides the histone binding motif in the Spt16-N terminus, the middle domains of both Spt16 (Spt16-M) and Pob3 (Pob3-M) have been shown to interact with H3/H4, suggesting that Spt16-N may work in conjunction with other H3/H4 binding domains, such as Spt16-M and Pob3-M, for histone binding in cells. Nonetheless, these results strongly indicate that the primary effect of Spt16-ΔN mutant on parental histone transfer is unlikely to be mediated by alterations in its interactions with H3/H4.

Subsequently, considering that both the *spt16-ΔN* and *mcm2–3A* mutants affect parental histone transfer to the lagging strand, we investigated whether the Spt16-N domain collaborates with Mcm2–7 during this process. Our findings revealed an interaction between FACT and the Mcm2–7 complex, with the Spt16-N domain playing a crucial role in mediating this interaction in cells using multiple assays. Finally, we found that the Spt16-N domain regulates the formation of FACT–H3/H4-Mcm2HBD ternary complex. *In vitro*, we found that Mcm2HBD does not bind FACT in the absence of histones. Interestingly, when Mcm2HBD domain binds H3/H4, a complex of Mcm2HBD–H3/H4–FACT forms. Under this condition, depletion of the Spt16-N domain has no apparent effects on the formation of Mcm2HBD–H3/H4–FACT complex. In contrast, if FACT binds H3/H4 first, deletion of Spt16 N-domain results in increased formation of Mcm2HBD–H3/H4–FACT complex. This result suggests that in addition to mediating the interaction between FACT and Mcm2–7 complex, the N-terminus of Spt16 also regulates the formation of Mcm2HBD–H3/H4–FACT complex. We suggest that the Spt16-N domain serves as a safeguard mechanism (Figures 5H and 6D). The intact wild-type FACT complex, when bound to parental H3/H4, does not release or expose parental H3/H4 for interaction with the Mcm2HBD. Instead, it awaits the establishment of a proper interaction between FACT–Mcm2–7 complex, likely mediated by the Spt16-N domain. Once this interaction is established, the FACT-bound histone H3/H4 is exposed for interaction with the Mcm2HBD, facilitating parental histone recycling. In the absence of the Spt16-N domain, FACT-bound H3/H4 becomes exposed, thereby mediating interaction with Mcm2HBD *in vitro*. However, this model cannot exclude the possibility that the mutant FACT*Δ*N -H3/H4 complex is not as stable as the WT FACT–H3/H4 complex. In *spt16-ΔN* cells, the proper interaction between the FACT and Mcm2–7 complex fails to occur. As a consequence, the exposed H3/H4 may be released to other histone chaperones, resulting in a noticeable reduction in the amount of H3/H4 bound to Mcm2 in *spt16-ΔN* cells. Consistent with this idea, we observed a substantial decrease in parental histone recycling at the leading strand in *mcm2–3A spt16-ΔN* double-mutant cells.

Furthermore, in addition to Mcm2–7, previous studies have demonstrated the mediation of the FACT–Tof1 interaction by the Spt16-N domain (33,34), raising the question of whether Tof1 and the FACT–Tof1 interaction contribute to parental histone transfer. Additionally, mass spectrometry analysis has revealed that, apart from the Mcm2–7 complex, the Spt16-N domain is essential for FACT’s interaction with several other replisome components, including the Mrc1–Tof1–Csm3 complex and the DNA Pol α complex. Therefore, FACT likely collaborates with Mcm2 and other potential involved replisome components to ensure efficient recycling and transfer of parental histones (Figure 6E). Together, these findings collectively support the notion that FACT likely cooperates with multiple replisome components, which act as co-chaperones, to form a ternary complex for parental histone recycling and transfer.

## Supplementary Material

gkad846_Supplemental_Files

## Data Availability

The raw sequence data reported in this paper have been deposited in the Genome Sequence Archive (41) in the National Genomics Data Center (42), China National Center for Bioinformation/Beijing Institute of Genomics, Chinese Academy of Sciences (GSA: CRA006313). The mass spectrometry proteomics data have been deposited to the ProteomeXchange Consortium via the PRIDE (54) partner repository with the dataset identifier PXD043392.

## Notes

Present address: Xuezheng Wang, Laboratory of Epigenome Biology, Systems Biology Center, Division of Intramural Research, National Heart, Lung, and Blood Institute, National Institutes of Health, Bethesda, MD, USA.
