# Supplementary material for: The N-terminus of Spt16 anchors FACT to MCM2–7 for parental histone recycling
Source: Nucleic Acids Res. 2023 Oct 18;51(21):11549–67. doi: 10.1093/nar/gkad846 (PMC10681723; doi:10.1093/nar/gkad846)
Supplement: gkad846_Supplemental_Files [file gkad846_supplemental_files.zip › 2023 0919 Supporting Information.pdf]

Supplementary Information for

The N-terminus of Spt16 anchors FACT to MCM2-7 for parental histone recycling

Xuezheng Wang[1,2#], Yuantao Tang[1#], Jiawei Xu[1], He Leng[1,2], Guojun Shi[1], Zaifeng Hu[1], Jiale Wu[1], Yuwen Xiu[2], Jianxun Feng[1*] and Qing Li[1,2*]

[1]State Key Laboratory of Protein and Plant Gene Research, School of Life Sciences and Peking-Tsinghua Center for Life Sciences, Peking University, Beijing, China 100871

[2]Academy for Advanced Interdisciplinary Studies, Peking University, Beijing, China 100871

*Corresponding authors:

Jianxun Feng

Email: fengjx@pku.edu.cn

Qing Li

Email: li.qing@pku.edu.cn

This PDF file includes:

Figures S1 to S10
Tables S1 to S5
Table S6 and S7 were uploaded separately
SI references

Fig. S1

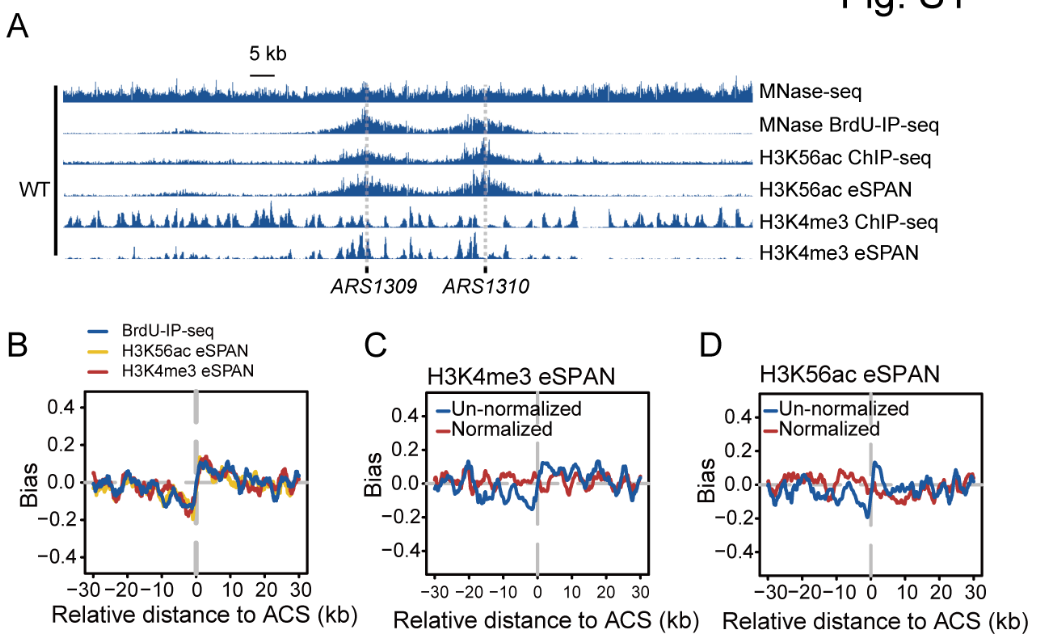

Figure S1. H3K4me3 and H3K56ac eSPAN analysis during S phase in wild-type cells.

(A) Snapshot of the MNase-Seq, MNase-BrdU-IP-Seq, H3K4me3 ChIP-Seq, H3K4me3 eSPAN, H3K56ac ChIP-Seq, and H3K56ac eSPAN datasets around replication origins *ARS1309* and *ARS1310* in wild-type (WT) cells. The scale bar represents a 5-kilo base pair (kbp) DNA region.

(B) The average bias of nascent DNA (BrdU-IP-Seq), H3K4me3 eSPAN peaks, and H3K56ac eSPAN peaks at early replication origins in wild-type cells. The average bias was calculated according to the Log_2 ratio of Watson/Crick reads.

(C and D) The average bias of H3K4me3 (C) and H3K56ac (D) eSPAN peaks at early replication origins in wild-type cells either unnormalized or normalized to the MNase-BrdU-IP-Seq dataset. The average bias was calculated as in Figure 1A according to the Log_2 ratio of Watson/Crick reads either unnormalized or normalized to the MNase-BrdU-IP-Seq dataset. The normalized eSPAN bias was used in subsequent analysis.

Fig. S2

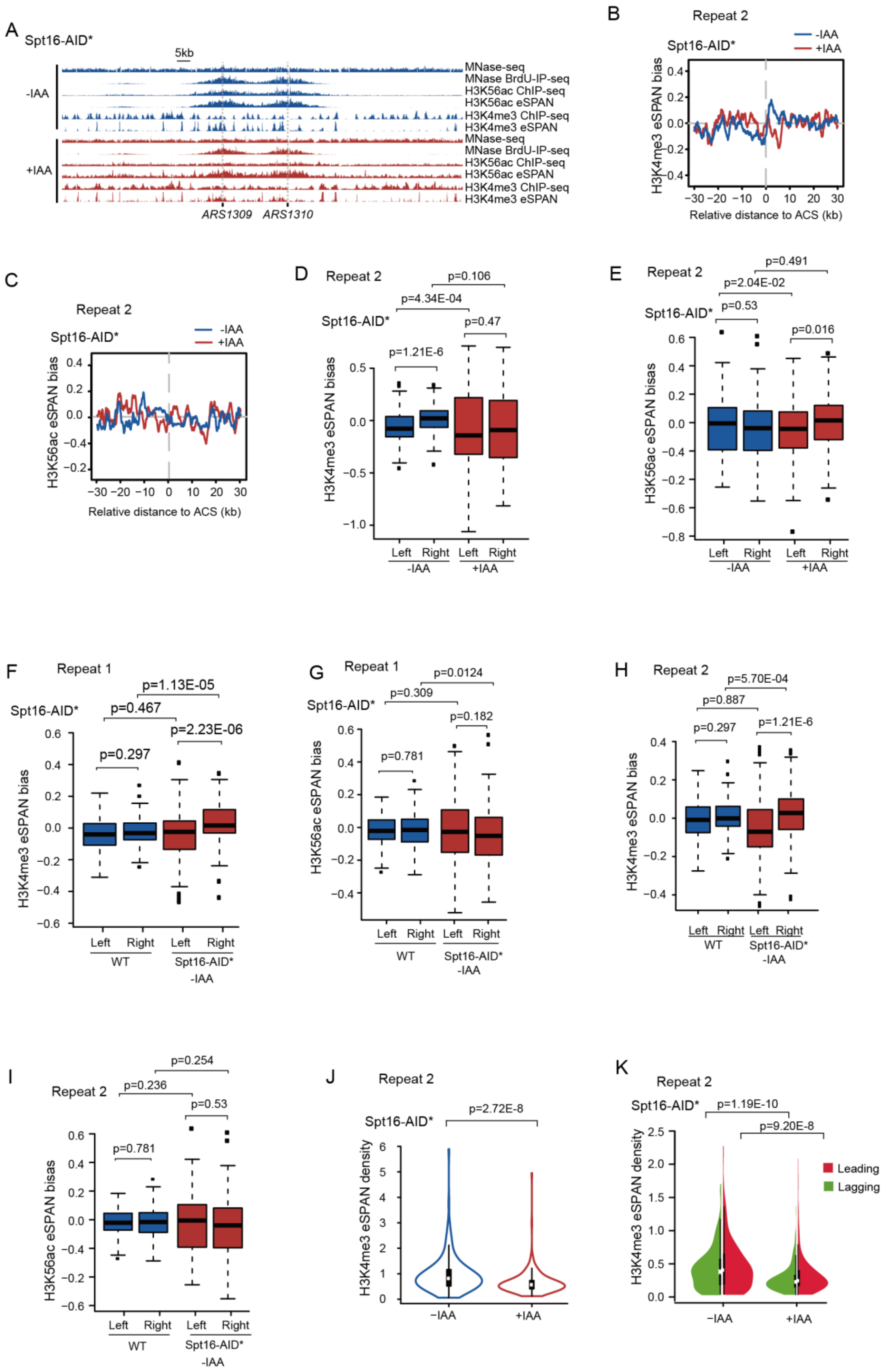
A
Spt16-AID*
5kb
-IAA
+IAA
MNase-seq
MNase BrdU-IP-seq
H3K56ac ChIP-seq
H3K56ac eSPAN
H3K4me3 ChIP-seq
H3K4me3 eSPAN
ARS1309
ARS1310
B
Repeat 2
Spt16-AID*
-IAA
+IAA
H3K4me3 eSPAN bias
Relative distance to ACS (kb)
C
Repeat 2
Spt16-AID*
H3K56ac eSPAN bias
Relative distance to ACS (kb)
D
Repeat 2
Spt16-AID*
p=0.106
p=4.34E-04
p=0.47
p=1.21E-6
H3K4me3 eSPAN bisas
Left Right Left Right
-IAA +IAA
E
Repeat 2
Spt16-AID*
p=0.491
p=2.04E-02
p=0.53
p=0.016
H3K56ac eSPAN bisas
Left Right Left Right
-IAA +IAA
F
Repeat 1
Spt16-AID*
p=1.13E-05
p=0.467
p=2.23E-06
p=0.297
H3K4me3 eSPAN bias
Left Right Left Right
WT Spt16-AID* -IAA
G
Repeat 1
Spt16-AID*
p=0.0124
p=0.309
p=0.182
p=0.781
H3K56ac eSPAN bias
Left Right Left Right
WT Spt16-AID* -IAA
H
Repeat 2
p=5.70E-04
p=0.887
p=0.297
p=1.21E-6
H3K4me3 eSPAN bias
Left Right Left Right
WT Spt16-AID* -IAA
I
Repeat 2
p=0.254
p=0.236
p=0.53
p=0.781
H3K56ac eSPAN bisas
Left Right Left Right
WT Spt16-AID* -IAA
J
Repeat 2
Spt16-AID*
p=2.72E-8
H3K4me3 eSPAN density
−IAA +IAA
K
Repeat 2
Spt16-AID*
p=1.19E-10
p=9.20E-8
H3K4me3 eSPAN density
Leading
Lagging
−IAA +IAA

Figure S2. Depletion of Spt16 results in defective in both parental histone recycling and new histone deposition.

(A) Snapshot of the MNase-Seq, MNase-BrdU-IP-Seq, H3K4me3 ChIP-Seq, H3K4me3 eSPAN, H3K56ac ChIP-Seq, and H3K56ac eSPAN datasets around replication origins *ARS1309* and *ARS1310* in cells with (+IAA) or without (-IAA) auxin-induced Spt16 degradation. The scale bar represents a 5-kilo base pair (kbp) DNA region.

(B and C) The average eSPAN bias of H3K4me3 (*C*) and H3K56ac (*D*) at early replication origins with (+IAA) or without (-IAA) auxin-induced Spt16 degradation. ACS: ARS (an autonomously replicating sequence) consensus sequence at replication origins. Similar results were obtained from at least two biological repeats; one representative experiment is shown.

(D and E) The box plot of eSPAN bias of H3K4me3(D) and H3K56ac (E) around early replication origin with (+IAA) or without (-IAA) Spt16 degradation. Left: the upstream of the origin (-30 kb~0 kb); Right: downstream of the origin (0~30 kb). Statistical significance was evaluated based on the Mann–Whitney U test.

(F and G) The box plot of eSPAN bias of H3K4me3 (F) and H3K56ac (G) around early replication origin in cells without Spt16-AID* degron system (WT) or with the Spt16-AID* degron system in the absence of the IAA treatment (-IAA). Statistical significance was evaluated based on the Mann–Whitney U test.

(H and I) The box plot of eSPAN bias of H3K4me3(H) and H3K56ac (I) around early replication origin in cells without Spt16-AID* degron system (WT) or with the Spt16-AID* degron system in the absence of the IAA treatment (-IAA). Statistical significance was evaluated based on the Mann–Whitney U test.

(J) The average eSPAN density of H3K4me3 at early replication origins with (+IAA) or without (-IAA) auxin-induced Spt16 degradation. Statistical significance was evaluated based on the Mann–Whitney U test.

(K) The average H3K4me3 eSPAN density on the leading (red) or lagging (green) strand at early replication origins with (+IAA) or without (-IAA) Spt16 degradation.

Statistical significance was evaluated based on the Mann–Whitney U test.

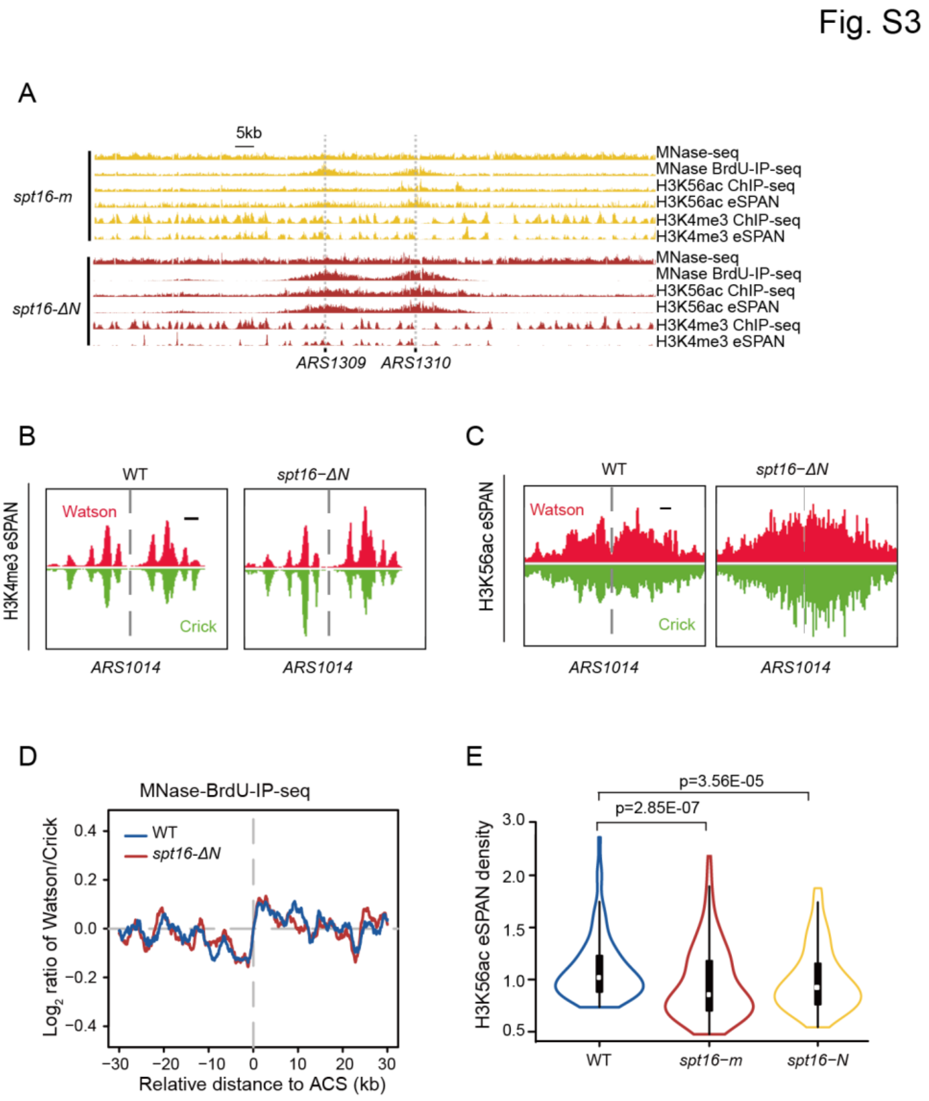

Figure S3. Mutations in Spt16 result in defective in both parental histone recycling and new histone deposition.

(A) Snapshot of the MNase-Seq, MNase-BrdU-IP-Seq, H3K4me3 ChIP-Seq, H3K4me3 eSPAN, H3K56ac ChIP-Seq, and H3K56ac eSPAN datasets around replication origins *ARS1309* and *ARS1310* in *spt16-m* and *spt16-ΔN* cells.

(B and C) Snapshot of H3K4me3 (D) and H3K56ac (E) eSPAN peaks in wild-type and *spt16-ΔN* cells at the region surrounding replication origin *ARS1014*. The scale bar represents a 2-kilo base pair (kbp) DNA region.

(D) The average bias of nascent DNA peaks in wild-type and *spt16-ΔN* cells at early

replication origins. The average bias was calculated according to the Log_2 ratio of Watson/Crick reads from the MNase-BrdU-IP-Seq dataset.

(E) The average H3K56ac eSPAN density in *spt16-m* and *spt16-ΔN* cells at early replication origins. Statistical significance was evaluated based on the Mann–Whitney U test.

Fig. S4

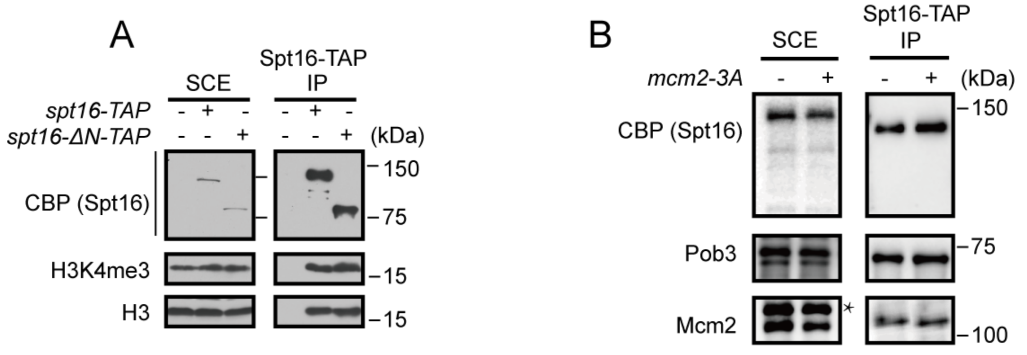

Figure S4. Spt16-ΔN does not reduce the histone binding ability of FACT.

(A) Spt16-N domain deletion does not reduce the binding of histone H3 to the Spt16-TAP complex. Tandem affinity purification (TAP)-tagged Spt16 was purified from wild-type and *spt16-ΔN* yeast cells. The co-purified proteins (IP) were resolved by SDS-PAGE and detected using the indicated antibodies. SCE: Soluble cell extracts.

(B) The *mcm2-3A* mutation does not affect the interaction between FACT and Mcm2. TAP-tagged Spt16 was purified from wild-type and *mcm2-3A* yeast cells. The co-purified proteins (IP) and soluble cell extracts (SCE) were resolved by SDS-PAGE and detected using the indicated antibodies. *Protein A in the TAP tag is recognized by the anti-Mcm2 antibody.

Fig. S5

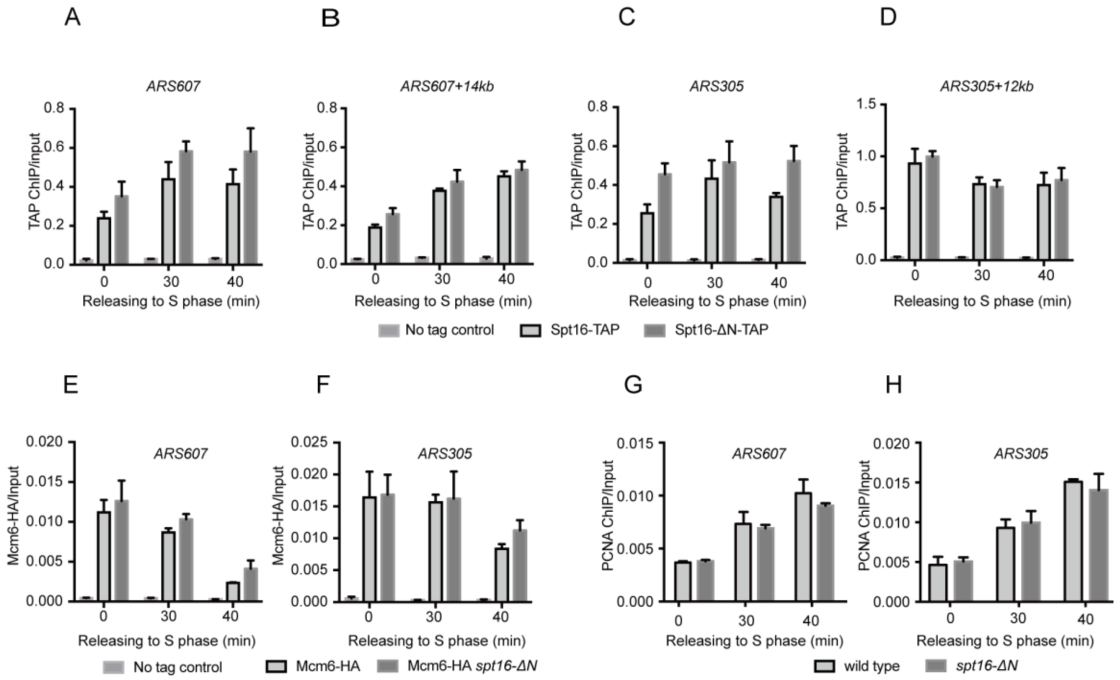

Figure S5. The Spt16-N domain deletion does not reduce the chromatin binding of FACT or several replisome components in the replicating regions.

(A–D) Analysis of the chromatin binding of TAP-tagged Spt16 in G1 and S phase around replicating regions by ChIP. Briefly, yeast cells expressing TAP-tagged wild-type (WT) or the Spt16-N domain deletion (Spt16ΔN) were synchronized at the G1 phase by the addition of α factor and then released into YPD for 30 min or 40min to allow for the entrance into the early S phase. After crosslinking with formaldehyde, cells were fragmented by sonication and subjected to Spt16-TAP ChIP using IgG beads. Quantitative real-time PCR was used to analyze ChIP and input DNA samples with primers against the early replication origins *ARS607* (A) and *ARS305* (C) and their corresponding distal regions *ARS607-14 kb* (B) and *ARS305-12 kb* (D). Error bars represent the standard error as calculated from three biological replicates.

(E and F) Analysis of the chromatin binding of HA-tagged Mcm6, a subunit of the MCM2-7 complex, around replicating regions in the G1 and S phase by ChIP. The experimental procedure was performed as described for Spt16-TAP ChIP, with the

exception of an anti-HA antibody being used for ChIP in strains expressing Mcm6-3-HA. Quantitative real-time PCR was used to analyze the ChIP and input DNA samples with primers against the early replication origins *ARS607* (*E*) and *ARS305* (*F*). Error bars represent the standard error as calculated from three biological replicates.

(G and H) Analysis of PCNA binding to chromatin in G1 and S phase by ChIP. The experimental procedure was performed as described for Spt16-TAP ChIP, with the exception of an anti-PCNA antibody being used for ChIP. Quantitative real-time PCR was used to analyze the ChIP and input DNA samples with primers against the early replication origins *ARS607* (G) and *ARS305* (H). Error bars represent the standard error as calculated from three biological replicates.

Fig. S6

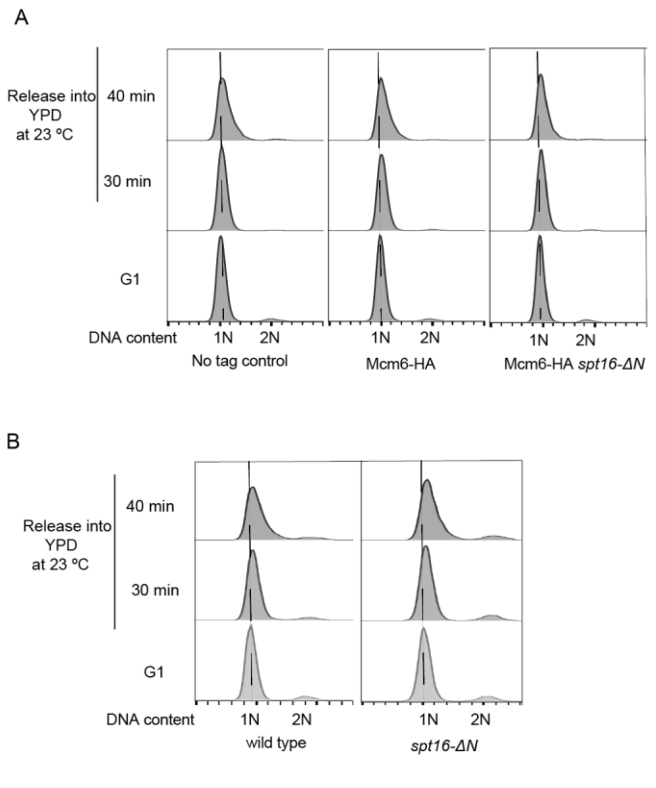

Figure S6. DNA content analysis of the ChIP samples in Figure S5. The experimental procedure was performed as described in Figure S5. Cellular DNA content was measured by flow cytometry with PI staining.

Fig. S7

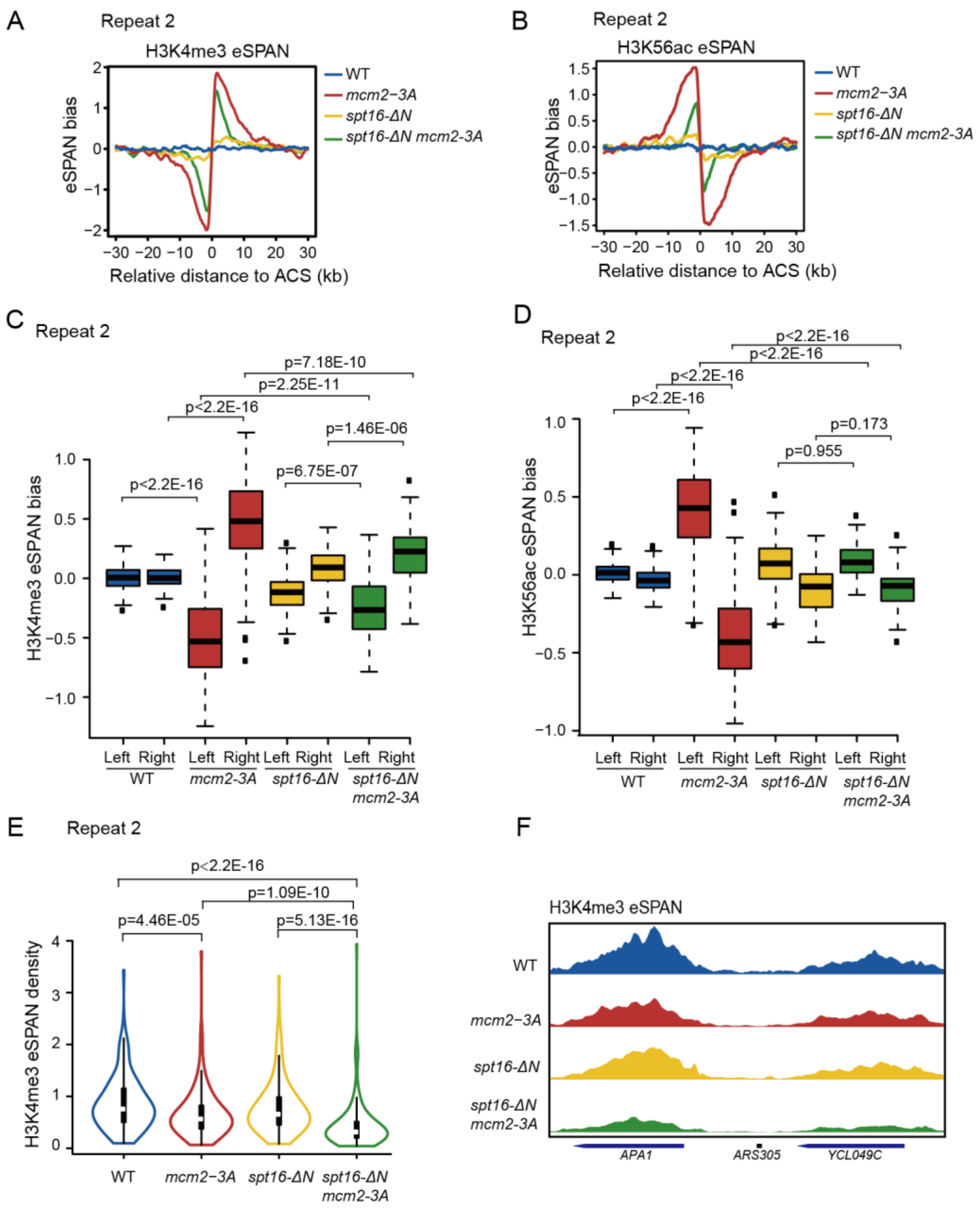

Figure S7. Parental histone transfer through Mcm2 is partially dependent on the Spt16-N domain.

(A and B) The average eSPAN bias of H3K4me3 (A) and H3K56ac (B) at early replication origins in wild-type (WT), *mcm2-3A*, *spt16-ΔN*, and *mcm2-3A spt16-ΔN* cells. ACS: ARS consensus sequence at replication origins. Similar results were obtained from at least two biological repeats; one representative experiment is shown.

(C and D) The box plot of eSPAN bias of H3K4me3(C) and H3K56ac (D) around early replication origin in wild-type (WT), *mcm2-3A*, *spt16-ΔN*, and *mcm2-3A spt16-ΔN* cell.

left: the upstream of the origin (-30 Kb~0 Kb); right: downstream of the origin (0~30 Kb). Statistical significance was evaluated based on the Mann–Whitney U test.

(E) The average H3K4me3 eSPAN density in wild-type (WT), *mcm2-3A*, *spt16-ΔN*, and *mcm2-3A spt16-ΔN* cells at early replication origins. Statistical significance was evaluated based on the Mann–Whitney U test.

(F) Snapshot of the H3K4me3 eSPAN signals around the *ARS305* early replication origin in wild-type, *mcm2-3A*, *spt16-ΔN*, and *mcm2-3A spt16-ΔN* cells.

Fig. S8

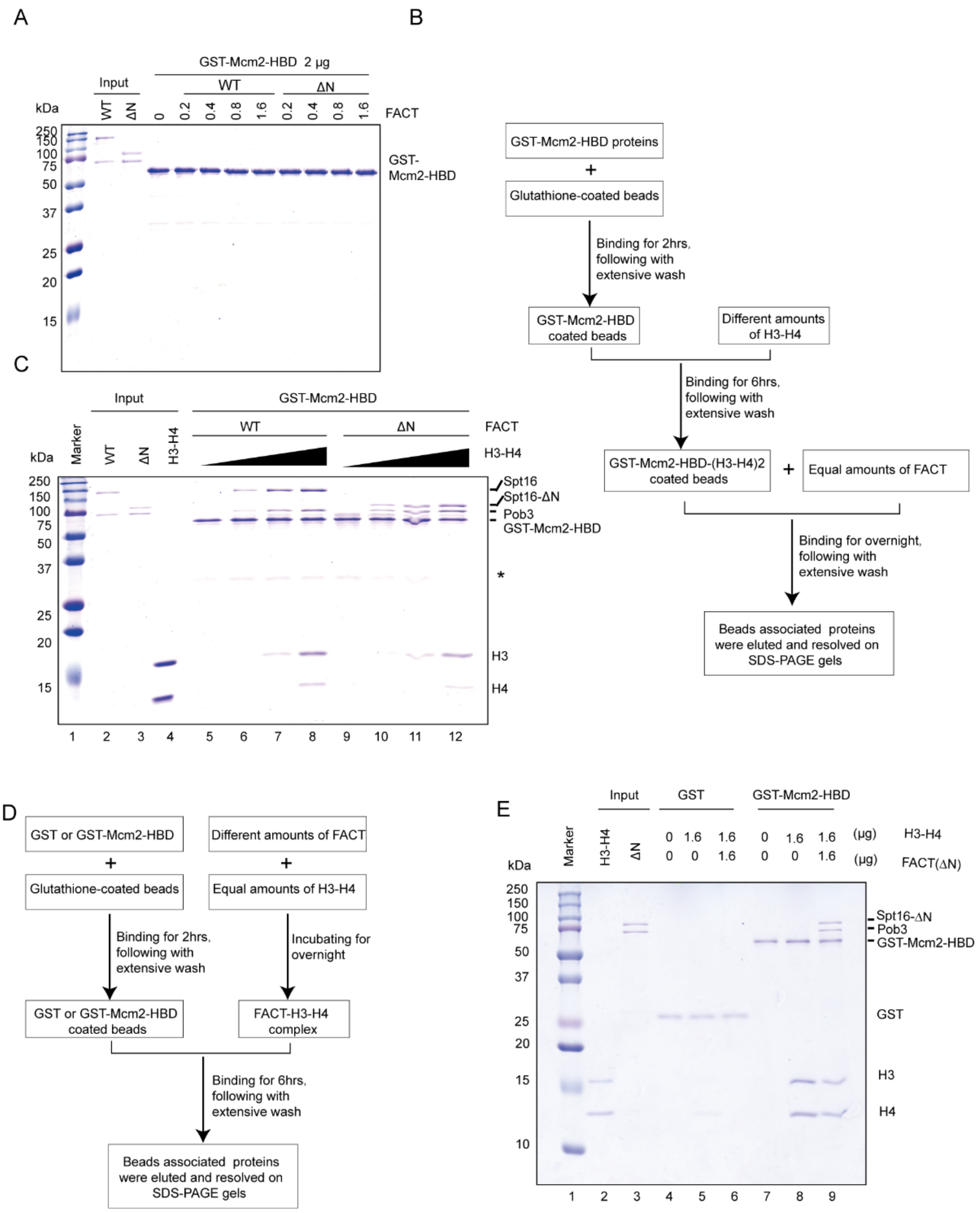

Figure S8. The Mcm2 HBD does not bind directly to FACT, and histone H3-H4 bridges this interaction *in vitro*.

(A) GST-pulldown assay of the GST-Mcm2 HBD with FACT. An increasing amount of FACT was incubated with 2 μg GST-Mcm2 HBD-coated beads, and the bound proteins were resolved by SDS-PAGE and visualized by CBB staining. Similar results were obtained from at least three independent experiments; one representative experiment is shown.

(B) Graphic outline of the experimental procedure for the GST-Mcm2 HBD pulldown assay. Briefly, the recombinant GST-Mcm2 HBD was first incubated with glutathione-coated beads to generate GST-Mcm2 HBD-coated beads and subsequently incubated with an increasing amount of histone H3-H4. The resulting GST-Mcm2-HBD–(H3-H4)$_2$ beads were then incubated with an equal amount of WT or Spt16ΔN FACT complex. After extensive washing of the beads, bound proteins were eluted, resolved by SDS-PAGE, and visualized by CBB staining.

(C) Histone H3-H4 bridges the interaction between the GST-Mcm2 HBD and FACT. The experiment was performed as described in (B). *Non-specific protein. Similar results were obtained from at least three independent experiments; one representative experiment is shown.

(D) Graphical outline of the experimental procedure for the *in vitro* histone capture assay.

(E) GST-Mcm2-HBD binds the Spt16-ΔN FACT–H3-H4 complex. Spt16-ΔN FACT complex was incubated overnight with recombinant histone H3-H4 to allow for the formation of the Spt16-ΔN FACT–H3-H4 complex. These proteins were then mixed with an equal amount of GST proteins- or GST-Mcm2 HBD proteins -coated beads and subjected to an *in vitro* pulldown assay. The recovered protein complex was resolved by SDS-PAGE and visualized by CBB staining. Similar results were obtained from at least two independent experiments; one representative experiment is shown.

Fig. S9

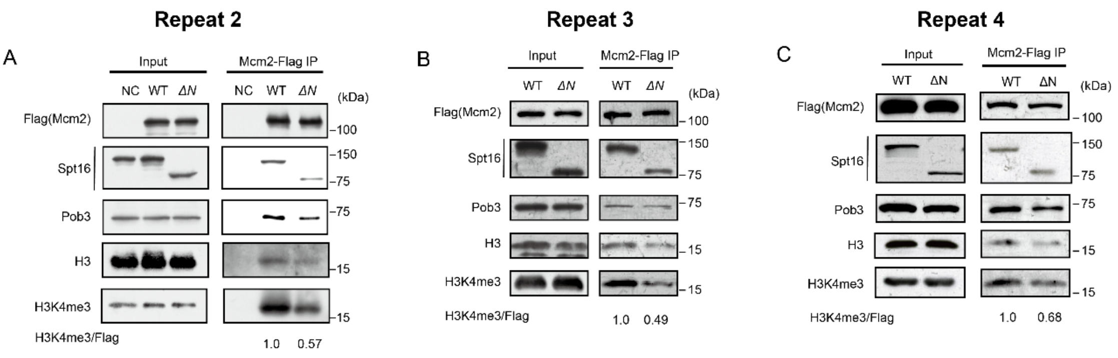

Figure S9. The Spt16-N domain is important for Mcm2 to bind histone H3 in cells. Flag-tagged Mcm2 was purified from wild-type (WT) and spt16-ΔN (ΔN) yeast cells, a strain without flag as control (NC). The co-purified proteins (IP) were resolved by SDS-PAGE and detected using the indicated antibodies. The intensity of H3K4me3 and Mcm2-Flag bands were quantitated using Image J and the ratio of H3K4me3/Flag was calculated. Four independent experiment results are shown.

Fig. S10

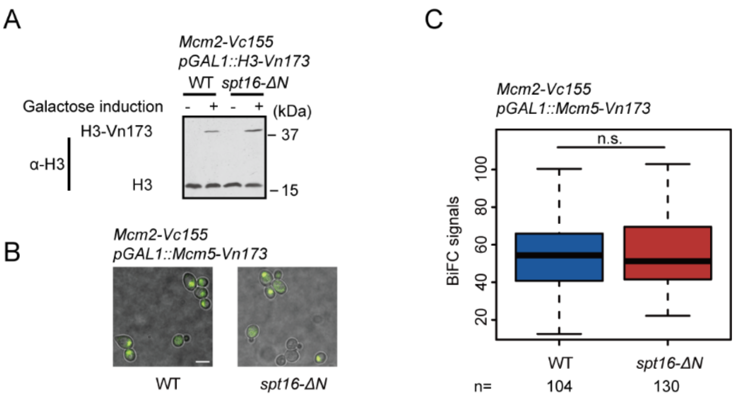

Figure S10. The effect of *spt16-ΔN* on the protein binding ability of Mcm2 in cells.

(A) Immunoblotting analysis of H3-Vn173 levels before and after galactose induction in wild-type (WT) and *spt16-ΔN* mutant cells.

(B) Spt16-N domain deletion does not affect the Mcm2–Mcm5 interaction in cells. BiFC intensity was imaged in wild-type (WT) and *spt16-ΔN* mutant cells expressing Mcm2-Vc155 and Mcm5-Vn173 constructs. The expression of Mcm5-Vn173 was

induced with 2% galactose for 2.5 h prior to imaging. The scale bar represents 5 μm. (C) Quantitated fluorescence intensities in (B*)*. Statistical significance was evaluated based on *t*-tests (n.s. no significance).

Table S1: Yeast strains used in this study

Strain	Genotype	Reference
LQY769 (W303-1A)	*MATa ade2-1 ura3-1 his3-11,15 trp1-1 leu2-3,112 can1-100*	(1)
LQY665	*MATa BrdU-Inc::TRP1*	(2)
LQY3509	*MATa BrdU-Inc::TRP1 spt16K692AR693A::natR*	This study
LQY4355	*MATa spt16-ΔN:: KanMX BrdU-Inc::TRP1*	This study
LQY5133	*MATa spt16-AID*-Flag:: hphNT pADH-OsTir1-9myc::URA3 BrdU-Inc::TRP1*	This study
LQY4127	*MATa mcm2-3A::hphNT BrdU-Inc::TRP1*	This study
LQY5135	*MATa mcm2-3A::hphNT spt16-ΔN::kan BrdU-Inc::TRP1*	This study
LQY24	*MATa spt16-ΔN-TAP::TRP1::KanMX*	This study
LQY113	*MATa spt16-TAP::TRP1*	(3)
LQY5345	*MATa spt16-TAP::TRP1 mcm2-3A::hphNT*	This study
LQY3713	*MATa mcm6-3HA::LEU2 BrdU-Inc::TRP1*	This study
LQY4701	*MATa mcm6-3HA::LEU2 spt16-ΔN::KanMX BrdU-Inc::TRP1*	This study
LQY4773	*MATa spt16-ΔN-TAP::TRP1::KanMX pol1-5Flag::NatR*	This study
LQY1304	*MATa Spt16-TAP::TRP1 mcm4-5Flag::NatR*	This study
LQY1309	*MATa spt16-m-TAP::TRP1::NatR mcm4-5Flag::NatR*	This study
LQY1296	*MATa Spt16-TAP::TRP1 mcm2-5Flag::NatR*	This study
LQY1308	*MATa spt16-m-TAP::TRP1::NatR mcm2-5Flag::NatR*	This study
LQY6285	*MATa spt16-ΔN-TAP::TRP1::KanMX mcm2-5Flag::NatR*	This study
LQY6286	*MATa spt16-ΔN-TAP::TRP1::KanMX mcm4-5Flag::NatR*	This study
LQY4561	*MATa spt16-eGFP::TRP1*	This study
LQY5191	*MATa spt16-ΔN-eGFP::TRP1::KanMX*	This study
LQY4372	*MATa mcm2-Vc155::HIS3*	This study
LQY6910	*MATa mcm2-Vc155::HIS3 spt16-ΔN:: KanMX*	This study

Table S2 Antibodies

reagent or resource	source	identifier
H3K4me3	Abcam	Cat#ab8580
H3	Abcam	Cat#ab1791
H3	EASYBIO	Cat#BE3015
BrdU	BD Biosciences	Cat#555627
HA	Roche	Cat#11583816001
Flag	Sigma	Cat#F1804
H4	EASYBIO	Cat#BE3194
CBP	EASYBIO	Cat#BE2068
PCNA	Laboratory of Zhiguo Zhang	
Spt16	Laboratory of Tim Formosa	
Mcm2	Laboratory of Huiqiang Lou	
H3K56ac	This lab	
Pob3	This lab	

Table S3 Reagents

reagent or resource	source	identifier
alpha factor	Chinese Peptide	Cat# MATE-001A
BrdU	Sigma	Cat#B5002
Protein G Sepharose resin	GE Healthcare	Cat#17061805
zymolyase 100T	MP	Cat#08320932
MNase	Worthington	Cat# LS004798
Chelex-100	Bio-rad	Cat#1422842
PMSF	Sigma	Cat# P7626
Benzamindine	Sigma	Cat# B6506
Pefobloc	Roche	Cat# 11429876001
DNase I	Sigma	Cat# DN25
IgG Sepharose resin	GE Healthcare	Cat#17096902
calmodulin affinity resin	Agilent	Cat#214303
Glutathione Sepharose resin	GE Healthcare	Cat#17513202
L-glutathione reduced	Sigma	Cat# G4251
nitrocellulose membrane	GE Healthcare	Cat#10600002
Bradford (Bio-Rad 5000205)	Bio-Rad	Cat#5000205
MinElute PCR purification kit	Qiagen	Cat#28006
Accel-NGS 1S Plus kit	Swift	Cat#10096

Table S4 Oligos used in this study

Name	Sequence (5'-3')
ARS607-F	tgccgcacgccaaacattgc
ARS607-R	cggctcgtgcattaagcttg
ARS305-F	agcaagaccggccagtttga
ARS305-R	gcactttgatgaggctctagcaa
ARS607-14kb-F	ctcttcatcactggagtcct
ARS607-14kb-R	cggctgtcatgccaagatgc
ARS305-12kb-F	gcggaagtctttgcaactgatatg
ARS305-12kb-R	tgcttgatttcttcgcagtattgg
BamHI-Mcm2-F	cgtggatccatgtctgataatagaagacgtagacg
EcoRI-TAA-MCM2-200	cgggaattcttattgtgttatccattccgagtaact

Table S5 Representative co-purified proteins with Spt16-TAP

Accession ID	Gene Name	Description	Fold Change	P Value
**P38132**	MCM7	DNA replication licensing factor MCM7	0.000146	0.006789
**P53840**	TOF1	Topoisomerase 1-associated factor 1	0.010695	0.00006
**Q04659**	CSM3	Chromosome segregation in meiosis protein 3	0.011596	0.000024
**P53685**	HST1	NAD-dependent protein deacetylase HST1	0.029875	0.054138
**P36124**	SET3	SET domain-containing protein 3	0.074715	0.000064
**P38121**	POL12	DNA polymerase alpha subunit B	0.084493	0.000005
**P38262**	SIF2	SIR4-interacting protein SIF2	0.085869	0.020591
**P53096**	HOS2	Probable histone deacetylase HOS2	0.086711	0.034359
**P25357**	SNT1	Probable DNA-binding protein SNT1	0.113962	0.000032
**P13382**	POL1	DNA polymerase alpha catalytic subunit A	0.150566	0.000091
**P40480**	HOS4	Protein HOS4	0.155898	0.000014
**P29469**	MCM2	DNA replication licensing factor MCM2	0.442003	0.006876
**P20457**	PRI2	DNA primase large subunit	0.540387	0.119197
**P29496**	MCM5	Minichromosome maintenance protein 5	0.561854	0.130811
**P30665**	MCM4	DNA replication licensing factor MCM4	0.717114	0.498182
**P14832**	CPR1	Peptidyl-prolyl cis-trans isomerase	0.825897	0.54969
**P24279**	MCM3	DNA replication licensing factor MCM3	0.938946	0.494863
**P32558**	SPT16	FACT complex subunit SPT16	0.999991	0.999825
**P53091**	MCM6	DNA replication licensing factor MCM6	1.052097	0.8212
**Q04636**	POB3	FACT complex subunit POB3	1.189839	0.083822

**P10363**	PRI1	DNA primase small subunit	1.461236	0.166605
**P02293**	HTB1	Histone H2B.1	1.640725	0.03908
**P47110**	POL32	DNA polymerase delta subunit 3	1.706837	0.11046
**P15436**	POL3	POL3 DNA polymerase delta catalytic subunit	1.777459	0.152553
**P61830**	HHT1; HHT2	Histone H3	1.948206	0.099529
**P24482**	DPB2	DNA polymerase epsilon subunit B	2.011613	0.045097
**P46957**	POL31	DNA polymerase delta small subunit	2.284657	0.02608
**P02309**	HHF1; HHF2	Histone H4	2.377479	0.052584
**P04911**	HTA1	Histone H2A.1	2.734123	0.014498
**P22336**	RFA1	Replication factor A protein 1	2.777856	0.099689
**P21951**	POL2	DNA polymerase epsilon catalytic subunit A	2.896438	0.18323
**P26754**	RFA2	Replication factor A protein 2	3.141646	0.129302
**P26754**	RFA2	Replication factor A protein 2	3.141646	0.129302
**P26755**	RFA3	Replication factor A protein 3	5.135973	0.067234
**Q04603**	DPB4	DNA polymerase epsilon subunit D	5.785308	0.056733

SI References:

1. Thomas BJ & Rothstein R (1989) Elevated recombination rates in transcriptionally active DNA. *Cell* 56(4):619-630.
2. Viggiani CJ & Aparicio OM (2006) New vectors for simplified construction of BrdU-Incorporating strains of Saccharomyces cerevisiae. *Yeast* 23(14-15):1045-1051.
3. Han J, *et al.* (2010) Ubiquitylation of FACT by the cullin-E3 ligase Rtt101 connects FACT to DNA replication. *Genes Dev* 24(14):1485-1490.
